# Germline polymorphisms in an enhancer of *PSIP1* are associated with progression-free survival in epithelial ovarian cancer

**DOI:** 10.18632/oncotarget.7047

**Published:** 2016-01-31

**Authors:** Juliet D. French, Sharon E. Johnatty, Yi Lu, Jonathan Beesley, Bo Gao, Murugan Kalimutho, Michelle J. Henderson, Amanda J. Russell, Siddhartha Kar, Xiaoqing Chen, Kristine M. Hillman, Susanne Kaufmann, Haran Sivakumaran, Martin O'Reilly, Chen Wang, Darren J. Korbie, Australian Ovarian Cancer Study Group, Australian Cancer Study, Diether Lambrechts, Evelyn Despierre, Els Van Nieuwenhuysen, Sandrina Lambrechts, Ignace Vergote, Beth Karlan, Jenny Lester, Sandra Orsulic, Christine Walsh, Peter A. Fasching, Matthias W. Beckmann, Arif B. Ekici, Alexander Hein, Keitaro Matsuo, Satoyo Hosono, Jacobus Pisterer, Peter Hillemanns, Toru Nakanishi, Yasushi Yatabe, Marc T. Goodman, Galina Lurie, Rayna K. Matsuno, Pamela J. Thompson, Tanja Pejovic, Yukie Bean, Florian Heitz, Philipp Harter, Andreas du Bois, Ira Schwaab, Estrid Hogdall, Susanne K. Kjaer, Allan Jensen, Claus Hogdall, Lene Lundvall, Svend Aage Engelholm, Bob Brown, James M. Flanagan, Michelle D. Metcalf, Nadeem Siddiqui, Thomas Sellers, Brooke Fridley, Julie Cunningham, Joellen M. Schildkraut, Ed Iversen, Rachel Palmieri Weber, Donal Brennan, Andrew Berchuck, Paul Pharoah, Paul Harnett, Murray D. Norris, Michelle Haber, Ellen L. Goode, Jason S. Lee, Kum Kum Khanna, Kerstin B. Meyer, Georgia Chenevix-Trench, Anna deFazio, Stacey L. Edwards, Stuart MacGregor

**Affiliations:** ^1^ QIMR Berghofer Medical Research Institute, Brisbane, Australia; ^2^ Department of Gynaecological Oncology and Centre for Cancer Research, The Westmead Institute for Medical Research, The University of Sydney, Westmead Hospital, Sydney, Australia; ^3^ Children's Cancer Institute Australia, Randwick, Australia; ^4^ Centre for Cancer Genetic Epidemiology, Department of Public Health and Primary Care, University of Cambridge, Cambridge, UK; ^5^ Cancer Research UK Cambridge Research Institute, Li Ka Shing Centre, Cambridge, UK; ^6^ Department of Health Sciences Research, Division of Biomedical Statistics and Informatics, Mayo Clinic, Rochester, MN, USA; ^7^ Australian Institute for Bioengineering and Nanotechnology, University of Queensland, Brisbane, Australia; ^8^ Peter MacCallum Cancer Centre, Melbourne, Australia; ^9^ Vesalius Research Center, VIB, Leuven, Belgium and Laboratory for Translational Genetics, Department of Oncology, University of Leuven, Leuven, Belgium; ^10^ Gynecologic Oncology, Leuven Cancer Institute, University Hospitals Leuven, Leuven, Belgium; ^11^ Women's Cancer Program at the Samuel Oschin Comprehensive Cancer Institute, Cedars-Sinai Medical Center, Los Angeles, CA, USA; ^12^ Department of Gynecology and Obstetrics, University Hospital Erlangen, Friedrich-Alexander University Erlangen- Nuremberg, Comprehensive Cancer Center Erlangen-Nuremberg, Erlangen, Germany; ^13^ Department of Medicine, Division of Hematology and Oncology, David Geffen School of Medicine, University of California, Los Angeles, CA, USA; ^14^ Division of Epidemiology and Prevention, Aichi Cancer Center Research Institute, Nagoya, Aichi, Japan; ^15^ Zentrum für Gynäkologische Onkologie, Kiel, Germany; ^16^ Departments of Obstetrics and Gynaecology, Hannover Medical School, Hannover, Germany; ^17^ Department of Gynecology, Aichi Cancer Center Central Hospital, Nagoya, Aichi, Japan; ^18^ Department of Pathology and Molecular Diagnostics, Aichi Cancer Center Central Hospital, Nagoya, Aichi, Japan; ^19^ Cancer Prevention and Control Program, Samuel Oschin Comprehensive Cancer Institute, Cedars Sinai Medical Center, Los Angeles, CA, USA; ^20^ Cancer Epidemiology Program, University of Hawaii Cancer Center, Hawaii, USA; ^21^ Department of Obstetrics and Gynecology, Oregon Health and Science University and Knight Cancer Institute, Oregon Health and Science University, Portland, OR, USA; ^22^ Department of Gynecology and Gynecologic Oncology, Dr. Horst Schmidt Kliniken Wiesbaden, Wiesbaden, Germany; ^23^ Department of Gynecology and Gynecologic Oncology, Kliniken Essen-Mitte, Essen, Germany; ^24^ Institut für Humangenetik Wiesbaden, Germany; ^25^ Danish Cancer Society Research Center, Unit of Virus, Lifestyle and Genes, Copenhagen, Denmark; ^26^ Molecular Unit, Department of Pathology, Herlev Hospital, University of Copenhagen, Copenhagen, Denmark; ^27^ Department of Gynecology, Rigshospitalet, University of Copenhagen, Denmark; ^28^ Department of Oncology, Rigshospitalet, University of Copenhagen, Denmark; ^29^ Department of Surgery and Cancer, Imperial College London, London, UK; ^30^ North Glasgow University Hospitals NHS Trust, Stobhill Hospital, Glasgow, UK; ^31^ Department of Cancer Epidemiology, Moffitt Cancer Center, Tampa, FL, USA; ^32^ Department of Biostatistics, University of Kansas Medical Center, Kansas City, KS, USA; ^33^ Department of Laboratory Medicine and Pathology, Mayo Clinic, Rochester, MN, USA; ^34^ Department of Community and Family Medicine, Duke University Medical Center, Durham, NC, USA; ^35^ Cancer Control and Population Sciences, Duke Cancer Institute, Durham, NC, USA; ^36^ Department of Statistical Science, Duke University, Durham, NC, USA; ^37^ Queensland Centre for Gynaecological Cancer, Brisbane, Australia; ^38^ Department of Obstetrics and Gynecology, Duke University Medical Center, Durham, NC, USA; ^39^ Centre for Cancer Genetic Epidemiology, Department of Oncology, University of Cambridge, Cambridge, UK; ^40^ Crown Princess Mary Cancer Centre and Centre for Cancer Research, The Westmead Institute for Medical Research, The University of Sydney, Westmead Hospital, Sydney, Australia; ^41^ Department of Health Science Research, Division of Epidemiology, Mayo Clinic, Rochester, MN, USA; ^42^ Institute of Human Genetics, Friedrich-Alexander-Universität Erlangen-Nürnberg, Erlangen, Germany

**Keywords:** epithelial ovarian cancer, progression free survival, genome-wide association study, PSIP1, chromosome conformation capture

## Abstract

Women with epithelial ovarian cancer (EOC) are usually treated with platinum/taxane therapy after cytoreductive surgery but there is considerable inter-individual variation in response. To identify germline single-nucleotide polymorphisms (SNPs) that contribute to variations in individual responses to chemotherapy, we carried out a multi-phase genome-wide association study (GWAS) in 1,244 women diagnosed with serous EOC who were treated with the same first-line chemotherapy, carboplatin and paclitaxel. We identified two SNPs (rs7874043 and rs72700653) in *TTC39B* (best P=7×10^−5^, HR=1.90, for rs7874043) associated with progression-free survival (PFS). Functional analyses show that both SNPs lie in a putative regulatory element (PRE) that physically interacts with the promoters of *PSIP1*, *CCDC171* and an alternative promoter of *TTC39B*. The *C* allele of rs7874043 is associated with poor PFS and showed increased binding of the Sp1 transcription factor, which is critical for chromatin interactions with *PSIP1*. Silencing of PSIP1 significantly impaired DNA damage-induced Rad51 nuclear foci and reduced cell viability in ovarian cancer lines. PSIP1 (PC4 and SFRS1 Interacting Protein 1) is known to protect cells from stress-induced apoptosis, and high expression is associated with poor PFS in EOC patients. We therefore suggest that the minor allele of rs7874043 confers poor PFS by increasing PSIP1 expression.

## INTRODUCTION

Ovarian cancer is the fifth leading cause of cancer deaths among women worldwide with an estimated 225,500 new cases annually [1]. Although ovarian cancer is among the most chemo-sensitive of solid tumors and generally shows a good initial response to platinum/taxane treatment and optimal debulking surgery, the disease will recur in 60-80% of women with advanced disease within five years [2, 3]. Considerable effort has been focused on identifying predictors of outcome at the somatic level, but less emphasis has been placed on the identification of germline predictors of outcome. We and others have used the candidate gene approach to identify ATP-binding cassette family members that might be associated with PFS [4, 5]. However, these findings have not been convincingly validated [5, 6].

Genome-wide association studies (GWAS) have been extremely successful at finding susceptibility loci for many different complex diseases [7], including multiple cancers [8]. The successful identification of loci associated with response to treatment could have profound clinical implications for individualizing anti-cancer treatment but there have been very few successful GWAS identifying loci associated with outcome for any cancer [9–16]. One factor that might explain this is that for most cancers there is considerable heterogeneity in the chemotherapeutic regimens used, which is likely to contribute to heterogeneity in treatment response [17–19]. In addition, it has been difficult to compile germline DNA and detailed treatment and clinical follow-up information on a sufficiently large number of patients to provide enough statistical power to detect loci associated with PFS or overall survival (OS).

In this study, we aimed to identify germline polymorphisms that influence response to first-line chemotherapy in patients with EOC. Based on previous *ex vivo* studies in lymphoblastoid cell lines derived from related family members that have shown moderately high heritability (0.21 to 0.7, depending on dose) for sensitivity to docetaxel [20] and cisplatin-induced cytotoxicity [21], we hypothesized that inter-patient variability in response to these drugs may be in part be explained by genetic variation that could be identified if we used a cohort of patients who had been uniformly treated. Therefore, we conducted the GWAS of PFS in ovarian cancer patients treated with carboplatin and paclitaxel, with the initial GWAS on 385 patients with high-grade, serous cancer (HGSC) and follow-up phases on serous EOC patients from ten studies from the Ovarian Cancer Association Consortium (OCAC).

We identified two rare SNPs that fall within a regulatory element within intron 2 of *TTC39B*. Chromatin conformation assays showed that the targets of the regulatory element are *PSIP1*, *CCDC171* and an alternative promoter of *TTC39B*. DNA-protein analyses indicated that the likely functional SNP is rs7874043, which alters Sp1 transcription factor binding, a factor that is critical for chromatin looping between the PRE and the *PSIP1* promoter. Furthermore, we show that silencing of PSIP1 significantly impaired DNA damage-induced homologous recombination function in ovarian cancer cell lines. According to KM-plotter (an online database linking expression to ovarian outcome in publicly available data), high expression of *PSIP1* is associated with poor PFS in ovarian cancer suggesting that altered *PSIP1* expression may be driving the association between the associated SNPs and outcome in EOC patients [22].

## RESULTS

### Four-Phase GWAS

**Figure 1:**
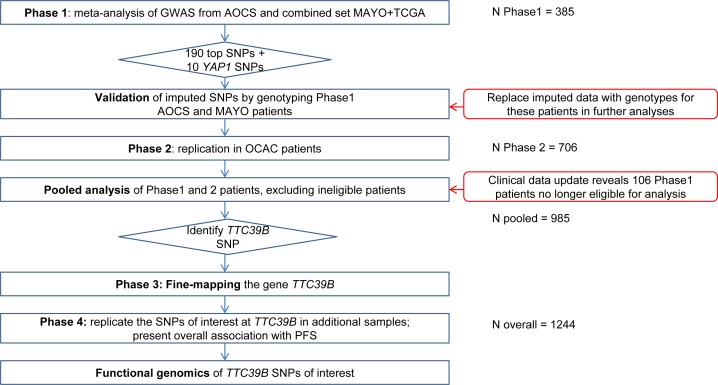
Study Design Overview of the study design for the identification of *TTC39B* SNPs using a four-phase GWAS of PFS in serous EOC patients.

We carried out a four-phase genome-wide association study of PFS in a total of 1,244 serous ovarian cancer patients who had debulking surgery and were uniformly treated with only carboplatin and paclitaxel as first-line therapy (Figure 1).

In Phase 1, we conducted a genome-wide scan on germline DNA from 385 patients from the Australian Ovarian Cancer Study (AOCS, *n* = 183), the Mayo Clinic (MAYO, *n* = 68) and The Cancer Genome Atlas (TCGA, *n* = 134) and performed a meta-analysis summarizing results from these cohorts (refer to Methods for details of genotyping and imputation). The Manhattan plot showing SNP association with PFS is presented in Supplementary Figure 1. We then prioritized 190 SNPs primarily ranked by P-value in Phase 1 for validation and further replication (Supplementary Table 1). We also included 10 SNPs in the gene *YAP1*, in light of its association with response to platinum-based chemotherapy in small-cell lung cancer patients [14], to test whether this finding would replicate in our study. These SNPs were also genotyped on Phase 1 AOCS and MAYO samples (DNA samples were not available for TCGA) so that we could replace imputed data with actual genotypes for these samples in subsequent analyses.

In Phase 2, we genotyped these 200 SNPs in 706 patients from 8 studies participating in OCAC. Following further data cleaning, we updated treatment details and other clinical information prior to Phase 2 analysis, which revealed 106 patients included in the Phase 1 GWAS who were no longer eligible for inclusion mainly due to the fact that these patients received additional agents or did not meet the dose requirement (Methods). Therefore, we performed a pooled analysis of 985 patients from both phases with these 106 patients excluded. This analysis identified two uncommon SNPs in strong linkage disequilibrium (LD), rs72700653 and rs7874043 (minor allele frequency (MAF) ∼ 1.85% and imputation r^2^ = 0.9) in intron 2 of *TTC39B*, most strongly associated with PFS in serous ovarian cancer patients (*P* = 3.5×10^−7^ and 3.6×10^−7^ for rs72700653 and rs7874043 respectively; Supplementary Table 1). Both SNPs were imputed with high quality (imputation quality score r^2^ = 0.81 in MACH [23]). None of the 10 tag SNPs in the *YAP1* gene were associated with PFS in these 985 patients (P > 0.05, Supplementary Table 1).

In Phase 3, we genotyped 38 tagSNPs, in addition to rs72700653 and rs7874043, in 985 OCAC samples to perform fine-mapping of the *TTC39B* locus. rs7874043 and rs72700653 remained the SNPs most associated with PFS at this locus, and the variants in moderate LD with rs7874043 showed consistent association with PFS (Supplementary Table 2). In Phase 4, we sought further replication of the association between these two variants and PFS in two additional cohorts, MAC (*n* = 26) and the clinical trial, ICON7 (*n* = 124) and additional samples from OCAC (*n* = 109). As there were only a small number of eligible cases in MAC, and both MAC and MAYO studies were recruited at the Mayo Clinic, we combined these two sets for analysis.

**Figure 2:**
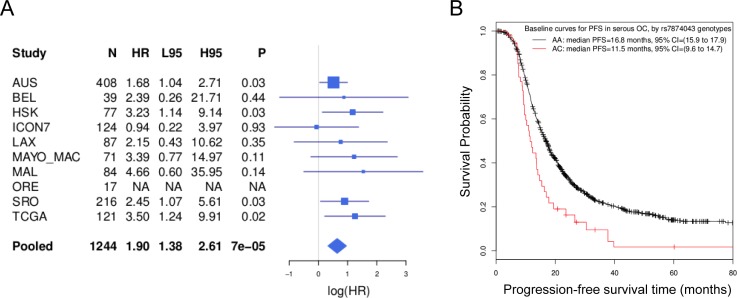
Association with PFS in serous EOC patients **A.** Associations between the *TTC39B* SNP rs7874043 and PFS in individual studies (rows denoted by study names), and the overall association pooling all samples together while stratifying for studies (the row denoted by “Pooled”). “HR” indicates the point estimates of hazard ratio. “L95” and “H95” represents its lower and upper 95% confidence intervals. “NA” indicates no minor allele was found in the eligible cases. The forest plot on the right is on the log scale. **B.** Baseline survival curves of the two genotypes (AA *vs* AC) of rs7874043 in a stratified Cox regression, assuming all other prognostic factors at mean values. Patients with CC genotypes were not observed due to the low minor allele frequency of rs7874043. The survival curves were truncated at 80 months as only a few events occurred after that.

To get an overall estimate of the hazard ratio, we pooled all available data from Phase 1, 2 (again excluding the ineligible patients) and 4 (*n* = 1244). Details of all the OCAC sites contributing to this study are given in Supplementary Table 3. This analysis showed that the minor allele of rs7874043 was associated with significantly worse PFS (HR = 1.90, 95% CI = 1.38 to 2.61, *P* = 7.3×10^−5^; Figure 2a). The median PFS in patients homozygous for the common allele of rs7874043 was 16.0 months (95% CI = 15.0 to 17.1), compared to 11.5 months (95% CI = 9.5 to 15.4) in heterozygous patients, without adjustment for covariates (log-rank test *P* = 0.0098); while the difference was 17.2 months (95% CI = 16 to 18.1) versus 11.5 months (95% CI = 9.6 to 14.7) when we assumed all prognostic factors at their mean values (Figure 2b, Supplementary Figure 2). The result of association between this SNP and PFS was similar when restricted to the high-grade serous patients at advanced disease stage (*n* = 1061, HR = 1.86, 95% CI = 1.33 to 2.6, *P* = 2.6×10^−4^). The median PFS was 14.8 months (95% CI = 14 to 15.8) for these patients with homozygous genotypes versus 11.0 months (95% CI = 9.3 to 14.1) for heterozygote patients, assuming mean covariates. The other SNP in high LD, rs72700653, despite a similar HR, had weaker association than rs7874043 due to more missing genotypes (HR = 1.91, 95% CI = 1.36 to 2.69, *P* = 2.2×10^−4^). We found attenuated associations with PFS in OCAC patients who were selected with no regard to chemotherapy (HR = 1.31, 95% CI = 1.04 to 1.66, *P* = 0.02; Supplementary Figure 3),

We also determined whether *TTC39B* SNPs were associated with OS. rs7874043 showed a significant, but weaker association with OS. The minor allele was also associated with worse OS (HR = 1.56, 95% CI = 1.09 to 2.23, *P* = 0.015). The median OS differences unadjusted for covariates were 46.3 months (95% CI = 43.2 to 49.9) for patients with homozygous genotypes versus 37 months (95% CI = 29.8 to 53.8) for heterozygous patients (log-rank test *P* = 0.048), and 48.7 months (95% CI = 45.4 to 55.3) versus 38.9 months (95% CI = 29.8 to 55.5) assuming mean covariates (Supplementary Figure 4).

### PFS-associated SNPs fall within a distal regulatory element of *PSIP1, CCDC171* and an alternative promoter of *TTC39B*

**Figure 3:**
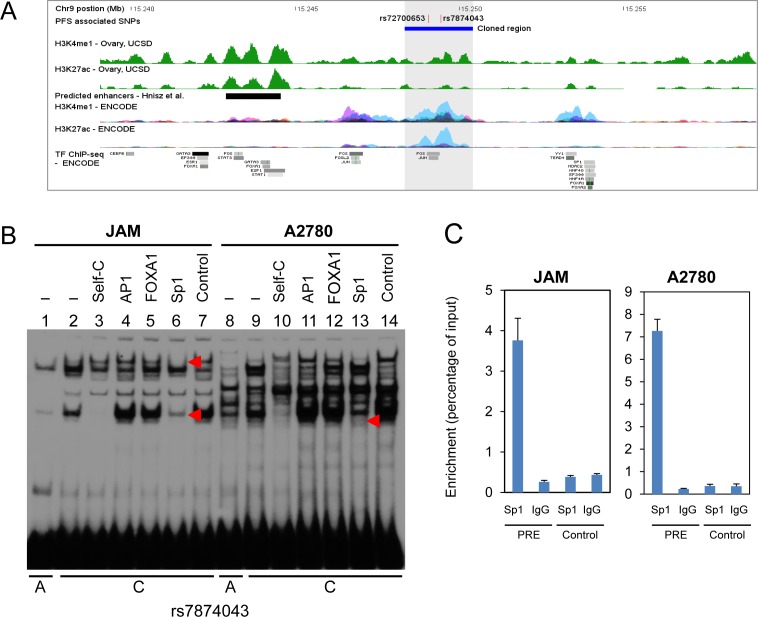
Chromatin structure and DNA-protein interactions surrounding the 9p22 PFS-associated SNPs **A.** Colored histograms denote histone modification ChIP-seq data from UCSD and ENCODE. Epigenetic marks for H3K4me1 and H3K27ac in ovary from UCSD and 7 cell types from ENCODE, and transcription factor ChIP-seq data from ENCODE are shown. The grey shaded region denotes the PRE containing SNPs rs72700653 and rs7874043. **B.** EMSA for oligonucleotides containing SNP rs7874043 with the A = common allele and C = minor allele as indicated below the panel, assayed using JAM and A2780 nuclear extracts. Labels above each lane indicate inclusion of competitor oligonucleotides at 30-fold molar excess: (−) no competitor (Lanes 1,2,8,9); Self-C allele (Lanes 3,10), AP1 (Lanes 4,11), FOXA1 (Lanes 5,12), Sp1 (Lanes 6,13) and a control sequence (Lanes 7,14; containing binding site for ATF, a TF not predicted to bind). The Sp1-containing complexes are indicated with red arrowheads. **C.** ChIP-qPCR on the PRE in JAM and A2780 cell lines. ChIP assays were performed with Sp1 antibodies or non-immune IgG, with a region 2.3kb upstream of the predicted Sp1-binding site (Control) used as a control for nonspecific binding. Graphs represent two biological replicates. Error bars denote SD.

Regulatory elements such as promoters and transcriptional enhancers/silencers can be identified by distinct chromatin marks. Tri-methylation of histone 3 lysine 4 (H3K4Me3) marks promoters, while mono-methylation (H3K3Me1) marks promoters and enhancers. ENCODE ChIP-seq data for H3K4Me1 from eight different cell lines covering the *TTC39B* locus revealed that both rs7874043 and rs72700653 fall within a putative regulatory element (PRE) marked by H3K4Me1 within intron 2 of *TTC39B* (Figure 3a). Transcription factor binding prediction indicated potential SNP-altered binding of Sp1, FOXA1 and AP1 (Supplementary Figure 5; [24]). We performed electrophoretic mobility shift assays (EMSAs) to assess binding of these transcription factors to the common and minor alleles of each of these variants and showed allele-specific protein binding for rs7874043 (Figure 3b; lanes 1, 2 and 8, 9). EMSAs using an Sp1 consensus oligonucleotide as competitor suggested that a strong higher mobility band and a weaker lower mobility band (in JAMs only) is likely to be Sp1 (Figure 3b, Supplementary Figure 6a). Using chromatin immunoprecipitation experiments we have shown that Sp1 is able to bind this site in JAM and A2780 ovarian cancer cell lines *in vivo* (Figure 3c). We also observed protein interaction at rs72700653, but found no difference in binding between alleles (Supplementary Figure 6b).

**Figure 4:**
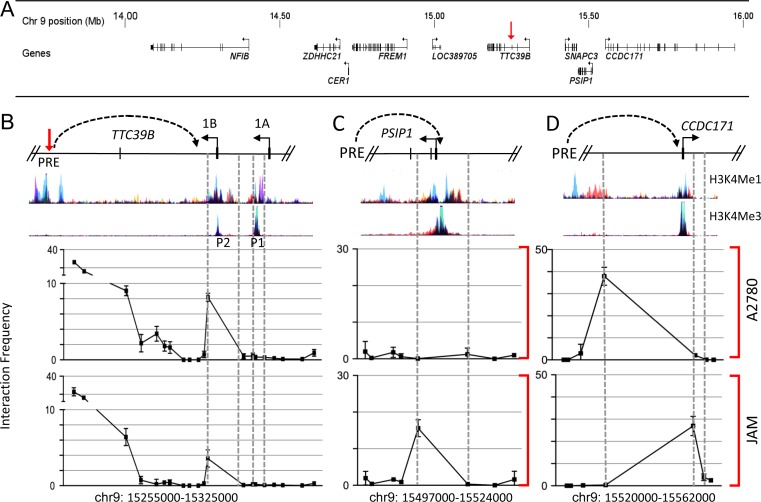
Chromatin interactions at 9p22 in ovarian cancer cell lines **A.** Physical map spanning 2Mb of the 9p22 region showing the position of all annotated genes assessed by 3C. The red arrowhead denotes *TTC39B*. **B.**–**D.** 3C interaction profiles between the PRE (containing rs72700653 and rs7874043) and (B) *TTC39B*, (C) *PSIP1* and (D) *CCDC171* promoter regions. 3C libraries were generated with either *Hin*dIII (B) or *Eco*RI (C and D), with the anchor point set at the PRE. A physical map of the region interrogated by 3C and relevant ENCODE histone modification data is shown above. A representative graph of three biological replicates is shown. Error bars denote SD.

To determine the likely target genes of the PRE, we performed chromosome conformation capture (3C) using an anchor primer within the restriction fragment encompassing the PRE and a series of primers within restriction fragments spanning all protein coding gene promoters within two megabases of the PRE (Figure 4a). The results showed that the PRE frequently interacts with an alternative (1B) promoter of *TTC39B* in both A2780 and JAM cells (Figure 4b). The PRE also frequently interacted with the *PSIP1* (also known as *LEDGF*) promoter, located approximately 260kb away, in JAM but not A2780 cells, and the *CCDC171* promoter, approximately 300kb away, in both JAM and A2780 cells (Figure 4c and 4d; Supplementary Figure 7). All chromatin interactions were confirmed by performing 3C with an independent restriction enzyme using anchor primers in the relevant gene promoters and a series of primers spanning the PRE (Supplementary Figure 8). No significant interactions were detected between the PRE and other flanking genes including *NFIB*, *ZDHHC21, CER1, FREM1* or *SNAPC3* (Supplementary Figure 9).

**Figure 5:**
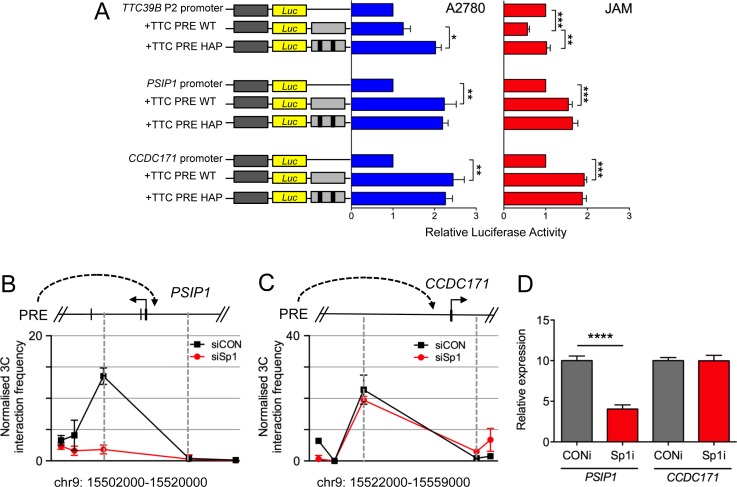
Evaluation of the function of rs72700653 and rs7874043 in ovarian cancer cell lines **A.** Luciferase assays comparing effect of minor alleles on the function of *TTC39B, PSIP1* and *CCDC171* promoters. The PRE was cloned upstream of *TTC39B*, *PSIP1* or *CCDC171* promoter-driven luciferase reporter constructs with rs72700653 and rs7874043 (PRE HAP) or without (PRE WT). A2780 or JAM ovarian cancer cells were transiently transfected with each construct and assayed for luciferase activity after 24h. Error bars denote SEM (*N* = 3). *P* values were determined with a two-tailed t test. *p < 0.05, **p < 0.01,***p < 0.001. Effect of siRNA knock-down of SP1 on 3-C interactions between the PRE with **B.** *PSIP1* and **C.** *CCDC171* promoter regions in JAM cells. 3C libraries were generated with *Eco*RI, with the anchor point set at the PRE. A physical map of the region interrogated by 3C data is shown above. A representative graph of three biological replicates is shown. Error bars denote SD. **D.** Effect of siRNA knock-down on gene expression levels of PSIP and CCDC171 in JAM cells. JAM cells were transiently transfected using Sp1 (siSp1) RNAi smartpools or nontargeting control (siCON) and assayed after 48 hours. Gene expression was measured by TaqMan and is given relative to *B-glucuronidase*. Error bars denote SEM (*N* = 3). *P* values were determined with a two-tailed t test. ****p < 0.0001.

Using luciferase reporter assays we demonstrated that the PRE acts as a strong transcriptional enhancer on the *PSIP1* and *CCDC171* promoters (Figure 5). Interestingly, the PRE had no significant effect on the *TTC39B* 1B promoter in A2780 cells and acted as a silencer in JAM cells suggesting that, depending on the cellular context, the PRE can act as an enhancer or silencer. To examine the effect of the SNPs on the activity of the PRE, we generated reporter constructs containing the minor alleles of both rs7874043 and rs72700653 (Figure 5, TTC PRE HAP). In A2780 cells, inclusion of the PRE-minor alleles significantly increased *TTC39B* 1B promoter activity and in JAM cells the minor alleles ablated the PREs silencer activity. In both cell lines, inclusion of the PRE-minor alleles had no additional effect on *PSIP1* and *CCDC171* promoter activity. While this appears to rule out a direct effect of these SNPs on transactivation of these promoters, Sp1 is reported to regulate chromatin looping and therefore the SNPs may be influencing the physical interactions between the PRE and target genes [25]. To address this question, we performed 3C analysis on JAM cells after Sp1 siRNA-mediated silencing and showed that the chromatin interaction between the PRE and *PSIP1* but not *CCDC171* was ablated (Figure 5b and 5c; Supplementary Figure 10). Consistent with this, the expression of *PSIP1*, but not *CCDC171*, decreased with Sp1 knockdown (Figure 5d).

### High expression of *PSIP1* and *CCDC171* is associated with PFS

**Figure 6:**
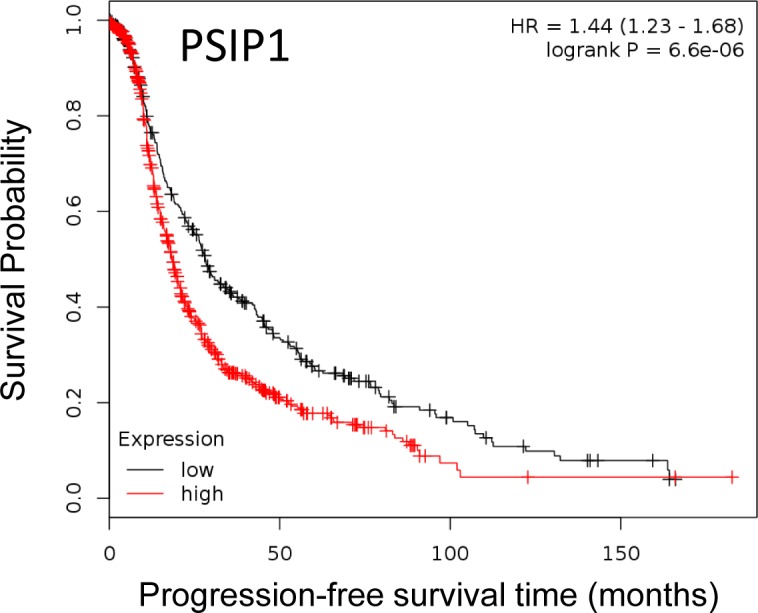
Kaplan-Meier curves of association between expression of PSIP1 with PFS in EOC Expression of *PSIP1* (Affymetrix probe 205961_s_at; log rank P = 6.6 × 10^−6^) and PFS in 1171 patients with serous and endometrioid EOC using the online tool KM-plotter [22]. High and low expression are defined as above and below the median.

We used Rapid Amplification of cDNA ends (RACE) to identify the transcript initiated from the 1B promoter of *TTC39B*, as there was none described. We identified a novel first exon of *TTC39B*, located ∼13 kb downstream of the canonical exon 1, with a successive exon structure similar to the published *TTC39B-202* (Supplementary Figure 11a). Negligible expression of this novel transcript was found in 18 ovarian cancer cell lines, whilst in 149 serous ovarian epithelial tumors from the AOCS low or minimal expression was observed (Supplementary Figure 11b and 11c). There was no association between expression of this transcript and PFS (HR = 0.7 (95% CI 0.38 - 1.25); *P* = 0.21 for the upper decile vs remaining patients); nor were expression levels associated with rs7874043 genotype (*P* = 0.22), but there were only six heterozygous carriers among the 142 tumors analyzed (Supplementary Figure 11c). However, analysis of 1171 epithelial ovarian tumors in KM-plotter [22], the online tool for survival-associated biomarkers, showed a strong association between high *PSIP1* expression and shorter PFS (Figure 6; HR = 1.44 (95% CI 1.23 - 1.68; *P* = 6.6 × 10^−6^ for comparison above and below median *PSIP1* expression). Since there were no data available in KM-plotter for *CCDC171*, we used the more limited TCGA serous ovarian cancer dataset and found that among the 68 patients with nil residual disease, high levels of *CCDC171* were associated with PFS (Supplementary Figure 12; HR 5.04 (95% CI 1.99 - 12.79); *P* = 0.001 for the upper decile vs remaining patients). However, this was not evident among 374 patients with any debulking status (HR 1.25 (95% CI 0.84 - 1.86); *P* = 0.266 for the upper decile vs remaining patients). In the TCGA dataset, expression of *PSIP1* was positively correlated with that of *CCDC171* and many other genes on the short arm of chromosome 9 (Supplementary Table 4). This appears to be largely because of co-amplification, as the correlations dropped noticeably upon correction for copy number.

### PSIP1 is required for RAD51 foci formation after DNA damage in ovarian cancer cell lines

**Figure 7:**
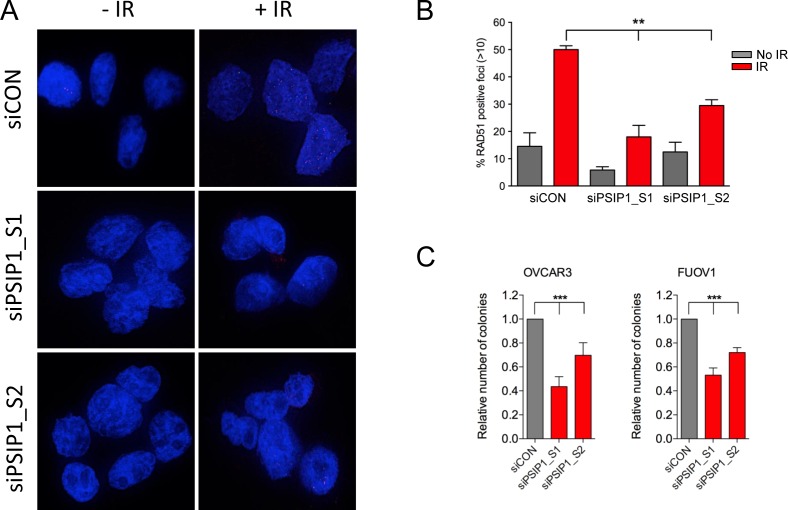
PSIP1 inhibition impaired DNA damage-induced homologous recombination function in ovarian cancer cell lines **A.** Representative images of the OVCAR3 cell line transfected either with nontargeting scramble control (siCON) or *PSIP1* (siPSIP1) RNAi for 48h, irradiated (IR) with 6 Gy and immunostained with anti-RAD51 (red) and DAPI (blue). **B.** Quantification of RAD51 positive foci after PSIP1 depletion alone and with 6 Gy IR. The percentage of cells with > 10 RAD51 foci were calculated. Error bars denote SEM (*N* = 2 with more than 50 cells were counted for each experiment). **C.** Effect of PSIP1 silencing on long-term colony formation in OVCAR3 and FUOV1 determined using crystal violet staining. *P* values were determined with a two-tailed t test. **p < 0.01,***p < 0.001.

PSIP1 is known to facilitate the resection step during homologous recombination mediated-repair and is required for RAD51 foci formation after DNA damage in a number of cancer cell lines [26]. Therefore, to assess this function in ovarian cancer we silenced *PSIP1* using siRNA in two high grade serous ovarian cancer cell lines, OVCAR3 and FUOV1 [27], which express relatively high levels of PSIP1 (Supplementary Figure 13a and 13b). We observed a significant reduction in DNA damage-induced Rad51 foci formation in both cell lines. Representative images and quantification are shown for OVCAR3 using two independent siRNA sequences (Figure 7a and 7b). Furthermore, exposure of OVCAR3 and FUOV1 cells to carboplatin and paclitaxel caused a moderate increase in PSIP1 levels consistent with PSIP1 being responsive to cellular stress, and in the case of carboplatin, potentially responsive to DNA damage (Supplementary Figure 13c). In long-term cell viability assays we also found that depletion of PSIP1 itself has significant effect on cell viability (Figure 7c) of ovarian cancer cell lines suggesting some level of dependency on PSIP1 levels for cell survival.

## DISCUSSION

We have carried out the first GWAS of PFS in European women diagnosed with EOC. We identified two SNPs in strong linkage disequilibrium (LD) in an intron of the *TTC39B* gene that were associated with worse PFS in patients with serous EOC. PFS in patients carrying the rare allele of rs7874043 is ∼5 months shorter than in patients carrying the common allele. In particular for patients with high-grade serous EOC at advanced stage who often have poor survival, the SNP is also associated with ∼4 months difference in PFS. Despite the large differences in PFS, these associations did not reach genome-wide significance (P < 5×10^−8^). We estimate that to reach genome-wide significance for the allele frequency of rs7874043, we would need twice as many serous EOC patients, depending on the true HR, with germline DNA samples and detailed clinical follow up data. We found attenuated associations with PFS in ovarian cancer patients who were selected with no regard to chemotherapy, reflecting either chance variation or that the effects of this SNP are specific to the treatment response. Furthermore, we found these SNPs had a significant, albeit weaker association with OS.

We have shown that the regulatory element, in which rs7874043 and rs72700653 lies, acts as a transcriptional enhancer on the *PSIP1* and *CCDC171* promoters, and that haplotype carrying the minor alleles of these SNPs enhances expression of the non-canonical *TTC39B* promoter. All three genes are located at 9p22.3, a region of chromosomal gain previously identified by comparative genomic hybridization in ovarian tumors as being associated with resistance to paclitaxel/carboplatin therapy [28]. The best characterized of the affected genes is *PSIP1* (PC4 And SFRS1 Interacting Protein 1), also known as *LEDGF* (Lens Epithelial Derived Growth Factor), which is a epigenetic reader recognizing H3K36 marks that preferentially associate with the internal coding areas of actively transcribed genes [29, 30]. PSIP1 is known to have oncogenic activity that controls a caspase-independent lysosomal cell death pathway [31], and can protect against cell death induced by many different stimuli including etopside, anthracyclines, docetaxel and oxidative stress [32]. PSIP1 is overexpressed in chemoresistant acute myelogenic leukemia and protects leukemic cells from apoptosis *in vitro* [33]. PSIP1 is thought to protect cells from stress-induced apoptosis by transcriptionally activating protective genes such as *HSP27* and *CRYAB* (alphaB-crystallin) [34]. More recently, PSIP1/LEDGF has been shown to regulate homologous recombination DNA repair pathway by guiding the tethering of Retinoblastoma binding protein 8 (RBBP8; also known as CtBP-Interacting Protein) to specific areas of chromatin (H3K36me3) in a DNA damage-dependent manner, providing a mechanism for its ability to protect cancer cells from DNA damage [35]. PSIP1 is also involved in HIV integration and is therefore a promising candidate for anti-retroviral therapy [36, 37].

Almost nothing is known about the function of CCDC171 (Coiled-Coil Domain Containing 171) except that it has been identified as an NRF1 target gene based on a Chip-Seq screen [38]. NRF1 plays a crucial role in maintenance of mitochondrial function and oxidative stress response. *TTC39B* (*c9orf52*) encodes a potential transmembrane protein and two GWAS for lipid levels have identified common SNPs in this locus associated with high density lipoprotein cholesterol (HDL-C) levels [39, 40]. This association was confirmed in a mouse model which showed that knockdown of *TTC39B* resulted in elevated levels of HDL-C [39]. These common SNPs are not strongly correlated with the rare SNPs we found to be associated with PFS (r^2^ < 0.1), and it is unknown whether they regulate the expression of the same novel *TTC39B* isoform that we identified. We cannot predict the function of this novel isoform but we have shown that it is not expressed at detectable levels in most ovarian cell lines, nor in approximately one-third of serous ovarian tumors.

Our data implicate Sp1 as a potential mediator of target gene(s) expression. Sp1 binds GC-rich DNA elements and regulates target genes by recruiting and complexing with transcription-associated proteins to activate or repress gene expression [41]. Notably, Sp1 is also able to mediate long-range activation of transcription through chromatin looping [30], and the *PSIP1* promoter CpG island contains Sp1-responsive sites [42]. We showed the minor *C* allele of rs7874043 preferentially binds Sp1 *in vitro*, and that Sp1 binds to a region encompassing rs7874043, *in vivo*. Consistent with the known function of Sp1 in chromatin looping, we showed that Sp1 silencing ablated chromatin looping with the *PSIP1* promoter and reduced *PSIP1* expression. We therefore suggest that the minor allele of rs7874043 enhances chromatin looping between the PRE and the *PSIP1* promoter to increase PSIP1 expression. Unfortunately, we were unable to identify any heterozygous cell lines for rs7874043, and therefore confirmation of these findings by evaluation of additional allele-specific effects was not possible in this study.

Although the absolute differences in PFS for carriers of the rare *TTC39B* alleles, compared to the common alleles, are quite large, the alleles are too rare to be responsible for much of the observed variation in PFS between affected women. Amongst the strengths of our study is the inclusion of only cases who had received standard first line treatment with carboplatin and paclitaxel, and the focus on clinically measurable PFS following first-line treatment, rather than on OS following exposure to multiple different drugs. Although we were able to obtain sufficient data in the population-based OCAC sites to conduct this study, it is much easier to do these studies in the context of clinical trials. Our study therefore emphasizes the importance of collecting germline DNA in clinical trials, and using them to detect biomarkers of response.

In conclusion, through a GWAS we have identified a SNP, rs7874043, as a very strong candidate for having a direct causal effect on PFS in ovarian cancer patients following first-line chemotherapy. We provide evidence that this SNP falls within a distal regulatory element that regulates several genes, including *PSIP1*, and show that high expression of *PSIP1* is associated with poor PFS in ovarian cancer patients. We observed a significant reduction in cell viability following PSIP1 inhibition, suggesting that PSIP1 is a potential target for therapeutic intervention in ovarian cancer as previously suggested for other cancers [31]. Moreover, like other cancer cell lines, transient silencing of PSIP1 in an ovarian cancer cell line significantly impaired DNA damage-induced RAD51 foci formation suggesting involvement of PSIP1 in the regulation of homologous recombination-mediated DNA repair. PSIP1 is involved in HIV integration, and so there is already some interest in developing specific inhibitors. Successful inhibition of PSIP1 may provide a novel approach to target ovarian cancer.

## MATERIALS AND METHODS

### Patient selection criteria

All participating studies received approval from the respective Institutional Ethics Boards. In order to study a homogeneous group of patients in Phase 1, we selected patients from AOCS, MAYO and TCGA based on the following criteria: the primary cancer sites described as ovarian, fallopian tube or primary peritoneal, invasive serous histology, collection of primary treatment response data completed at the time of patient selection, high grade (grade 2 or 3), FIGO stage III or IV. Similar patient selection criteria were applied to OCAC studies in the following phases, except that the patients with low grade and low stage were also included. To improve cost efficiency, we preferentially genotyped AOCS patients with extreme phenotypes in Phase 1 (Supplementary Methods).

We observed substantial heterogeneity among treatments that patients in OCAC received, with more than 80 different chemotherapy drug combinations, dosage levels and schedules used for first-line chemotherapy, so we further selected patients who were treated with only three weekly paclitaxel and carboplatin as first-line therapy. Among these patients, the majority received > 4 cycles of paclitaxel at a dose of 135 to 175 mg/m^2^ and carboplatin at AUC 5 to 7), while dosage for some patients was unknown.

Prior to commencing data analysis in Phase 2, we obtained updated clinical data, which revealed that 91 AOCS and MAYO patients included in Phase 1 no longer met the criteria we applied because they had received agents in addition to carboplatin/paclitaxel, or < 4 cycles of paclitaxel and carboplatin. Other data updates on residual disease and other clinical features meant that an additional 15 cases were no longer eligible for the analysis. We therefore excluded these cases from further analyses. All the following analyses were based on the most up-to-date clinical data. To summarize, we analyzed a total of 1244 cases who had received standard chemotherapy plus an additional 1346 cases who had received non-standard chemotherapy for the analyses selected with no regard to chemotherapy. The details of their treatment is provided in the Supplementary Table 5.

### Genotyping and Imputation

AOCS, MAYO and TCGA patients were genotyped using Illumina HumanOmni1-Quad arrays, HumanHap 610 arrays and Human1M arrays, respectively. We applied the following quality control steps for all three sets of GWAS data separately: 1) removing samples with > 10% missing genotypes; 2) excluding any SNP with less than 1% minor allele frequency (MAF); 3) excluding any SNP that failed the Hardy-Weinberg Equilibrium (HWE) test at the significance level of 5e-6; 4) excluding the SNPs with MAF > 5% when per SNP no-call rate > 5%, and those with MAF < 5% when per SNP no-call rate > 1%. We then assessed the cryptic relationship between the sample pairs using the ‘—genome’ command in PLINK [43]. The proportion of identical by descent (IBD) was estimated from the cleaned whole genome data, and then either of the paired IDs showing high levels of IBD sharing (PI_HAT > 0.2) was dropped. Patients who have been recruited in both MAYO and TCGA were identified using this approach and were included in the analyses only once. Following the check of cryptic relationships, we assessed potential population stratification using a Principal Components Analysis (PCA) algorithm EIGENSTRAT [44]. The HapMap 3 and GenomeEUTwin [45] individuals were used as a reference panel in the calculation of the principal components, and the current samples were projected into the background of reference population. We used 6 standard deviations (SDs) as the cut-off to identify ancestral outliers, which were removed from further association analysis. After these data cleaning, 183 AOCS patients (766,728 SNPs) and combined 68 MAYO and 134 TCGA patients (525,792 SNPs) were included in Phase 1 analysis.

To improve array comparison, we inferred the missing genotypes for the initial GWAS samples with the reference of the CEU samples from the 1000 Genomes pilot 1 data (June, 2010 release) using MACH 1.0 [23]. We had 385 samples on a common set of 297,906 SNPs without strand ambiguity (i.e. A|T or C|G genotypes) for imputation. We have imputed up to 6.86 million SNPs, with 88.7% (∼6 million) SNPs obtaining reasonable imputation quality (imputed R^2^ > 0.3).

We used the Sequenom MassARRAY iPLEX platform for genotyping in Phase 2-4, using previously described methods and quality control measures [46]. ‘Tag SNP Picker’ option at the HapMap web site was used to pick 45 SNPs which captured 187 *TTC39B* variants (MAF > 5%, in r^2^ > 0.5 with tag SNPs) from HapMap CEU set. 40 SNPs among this tag-SNP list were successfully genotyped and passed QC.

### Progression-free survival and overall survival

PFS was defined as the time interval between the date of histologic diagnosis and the first confirmed sign of disease recurrence, or progression (Supplementary Table 3). As a related survival trait, OS was defined as the time interval from date of diagnosis to time of last follow-up or time to death from any cause. To control for ascertainment bias, prevalent cases (with an interval > 12 months between the date of histological diagnosis and DNA collection) were excluded from analysis. There were a small number of cases who died without any reported evidence of progression (*N* = 17), and for them we applied right censoring to PFS at the time of last assessment.

### Statistical analysis

The allelic association with PFS or OS was assessed in a Cox Proportional Hazards (CPH) model, adjusting for potential site differences and prognostic factors of grade (low vs high), stage (4 levels), residual disease (nil vs any) and age of diagnosis (specific to the analysis of OS). We tested the proportional hazards assumption for the adjusted variables and stratified by those that violated the assumption. In most analyses, we found that study site was the major variable violating the assumption; therefore, we fitted sites as strata in the model. By fitting strata, we assumed that there were baseline level differences in PFS between the patients from different sites but no difference in the hazard ratio conferred by the SNP being tested.

The data from Phase 1 MAYO and TCGA sets were combined for analysis while stratifying for site differences, because, unlike those from AOCS, they were not selected for extreme phenotypes. To increase the statistical power in Phase 1, we performed a meta-analysis of the results from the AOCS and the combined MAYO and TCGA set using an inverse-variance weighting approach. When performing pooled analyses in Phase 2-4, we pooled data from all studies while stratifying by study sites.

### Cell lines

Human ovarian carcinoma cell lines A2780, JAM, OVCAR3 and FUOV1 were grown either in RPMI medium or DMEM/F12 with 10-20% FCS and antibiotics. Cell lines were maintained under standard conditions, routinely tested for *Mycoplasma* and identity profiled with short tandem repeat markers.

### Electrophoretic mobility shift assays (EMSAs)

EMSAs were carried out as previously described [47], except that oligonucleotides were detected using a Chemiluminescent Nucleic Acid Detection Module kit (Cat no 89880, Thermo Scientific). Oligonucleotide sequences used in the assays are listed in Supplementary Table 6. Competitor oligonucleotides were used at 30-fold molar excess.

### In silico prediction of transcription factor binding sites

Prediction of transcription factor binding sites was performed using the AliBaba 2.1 program (http://www.generegulation.com/pub/programs/alibaba2/index.html) [24].

### Chromatin immunoprecipitation (ChIP) qPCR

Sp1 ChIP-qPCR (Sp1; D4C3 rabbit monoclonal, Cell Signalling) assays were conducted as described previously [48] with a sheared fragment size of 300 bp to 1 kb. For qPCR, 1 μl from 30 μl of DNA extract was used. Primers are listed in Supplementary Table 6.

### Chromatin conformation capture (3C)

3C libraries were generated using *Hin*dIII, *Eco*RI or *Bgl*II as described previously [49]. 3C interactions were quantitated by real-time PCR (qPCR) using primers designed within restriction fragments (Supplementary Table 6). All qPCRs were performed on a RotorGene 6000 using MyTaq HS DNA polymerase with the addition of 5 mM of Syto9, annealing temperature of 66°C and extension of 30sec. 3C analyses were performed in three independent experiments with each experiment quantified in duplicate. BAC clones (RP11-746M21, RP11-940C5, RP11-356J15, RP11-728G24) covering the 9p22 region were used to create artificial libraries of ligation products in order to normalize for PCR efficiency. Data were normalized to the signal from the BAC clone library and, between cell lines, by reference to a region within *GAPDH*. All qPCR products were electrophoresed on 2% agarose gels, gel purified and sequenced to verify the 3C product.

### Plasmid construction and luciferase assays

The *TTC39B, PSIP1* and *CCDC171* promoter-driven luciferase reporter constructs were generated by inserting PCR-generated promoter fragments into the multiple cloning site (MCS) of pGL3-Basic. A 2.2 kb fragment containing the PRE was inserted into the *Bam*HI and *Sal*I sites downstream of luciferase. The minor alleles of rs72700653 and rs7874043 were introduced into promoter and PRE constructs by overlap extension PCR. All constructs were sequenced to confirm variant incorporation (AGRF, Australia). Primers used to generate all constructs are listed in Supplementary Table 6. A2780 and JAM ovarian cancer cells were transfected with equimolar amounts of luciferase reporter plasmids and 50ng of pRLTK using Lipofectamine 2000. The total amount of transfected DNA was kept constant per experiment by adding carrier plasmid (pUC19). Luciferase activity was measured 24 hours post-transfection using the Dual-Glo Luciferase Assay System on a Beckman-Coulter DTX-880 plate reader. To correct for any differences in transfection efficiency or cell lysate preparation, *Firefly* luciferase activity was normalized to *Renilla* luciferase. The activity of each test construct was calculated relative to promoter constructs, the activity of which was arbitrarily defined as 1.

### siRNA silencing

*Sp1* (L-026959-00) and non-targeting (D-001810-10-20) ON-TARGET*plus* SMARTpool, *PSIP1* (J-015209-05-0005 and J-015209-06-0005) and non-targeting (D-001810-01-05) siRNAs for were purchased from Thermo Scientific. For siRNA silencing, JAM cells were transfected with 25 nM of either *Sp1* or non-targeting siRNAs using Lipofectamine 2000 and lysates prepared for 3C after 48 hours.

### Taqman expression assays

JAM total RNA was extracted using Trizol (Life Technologies). Residual DNA contaminants were removed by DNAse treatment (Ambion) and complementary DNA was synthesized using random primers as per manufacturers' instructions (Life Technologies). All qPCRs were performed on a RotorGene 6000 (Corbett Research) with TaqMan Gene Expression assays (Hs01045711_g1 for Sp1, Hs00916521_m1 for *PSIP1* and Hs00411735_m1 for *CCDC171*) and TaqMan Universal PCR master mix. All reactions were normalized against *β-glucuronidase* (Catalogue No. 4326320E).

### RAD51 foci formation

OVCAR3 cells were reverse transfected with 10nM of siRNAs targeting *PSIP1* (siPSIP1) described above for 24 h and later seeded on the 0.1% poly-l-lysine coated coverslips followed by second reverse transfection for additional 24 h. To determine RAD51 foci accumulation, cells were irradiated (IR) with 6 Gy (^137^Cesium) and analyzed 6 h after irradiation as described previously [50].

### Colony formation assays

48 h after siRNA transfection, 10,000-20,000 cells were seeded in 24 well plates and incubated for additional 7 days to determine colony viability. The colonies were fixed with 0.05% crystal violet for 30 minutes, washed and quantified for crystal violet intensity after destaining using Sorenson's buffer (0.1 M sodium citrate in 50% Ethanol, pH 4.2) at 590 nM absorbance using PowerWave HT Microplate Spectrophotometer (BioTeK, USA).

## SUPPLEMENTARY MATERIAL FIGURES AND TABLES


