# Supplementary material for: Germline polymorphisms in an enhancer of PSIP1 are associated with progression-free survival in epithelial ovarian cancer
Source: Oncotarget. 2016 Jan 31;7(6):6353–68. doi: 10.18632/oncotarget.7047 (PMC4872719; doi:10.18632/oncotarget.7047)
Supplement: Supplementary file 1 [file oncotarget-07-6353-s001.pdf]

Germline polymorphisms in an enhancer of *PSIP1* are associated with progression-free survival in epithelial ovarian cancer

Supplementary Material

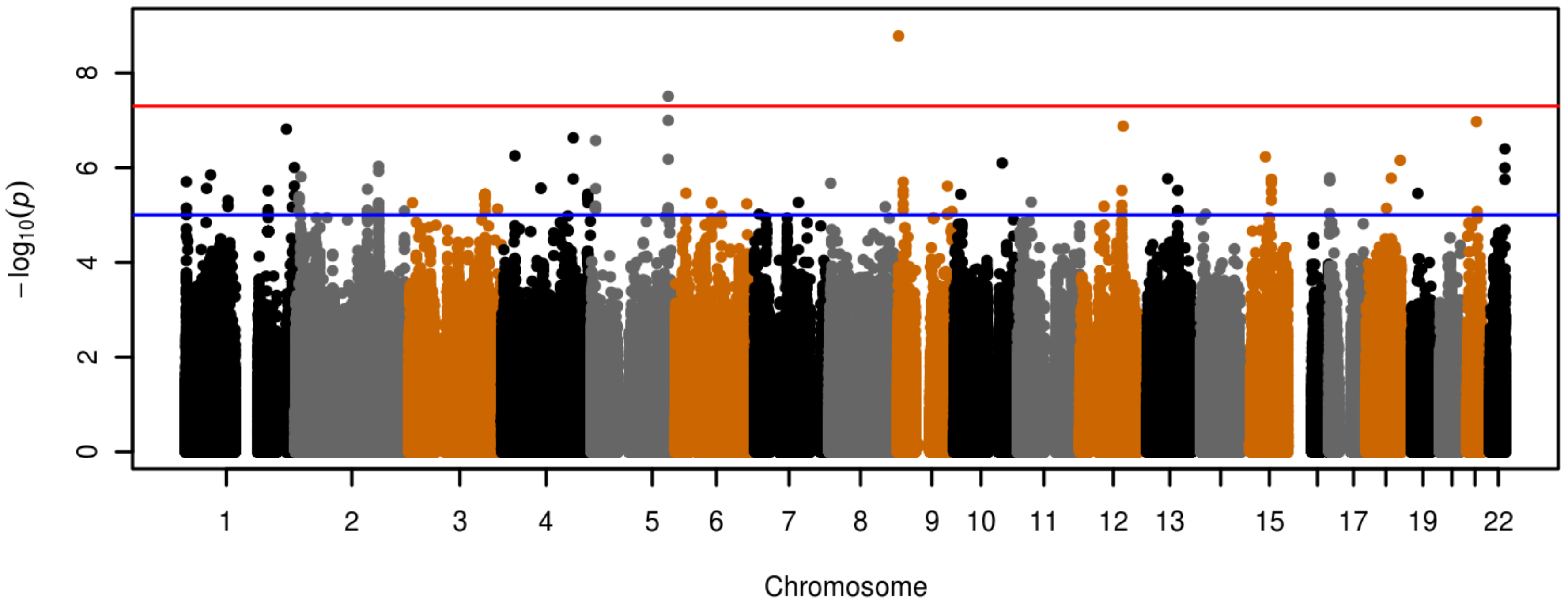

Supplementary Figure 1. **Manhattan plot of SNPs associated with progression-free survival (PFS) in Phase 1.** Association results ($-\log_{10}$ P-values) are plotted for each chromosome. The red line marks the genome-wide significance (P=5x10^{-8}) and the blue line marks the suggestive significance (P=1x10^{-5}).

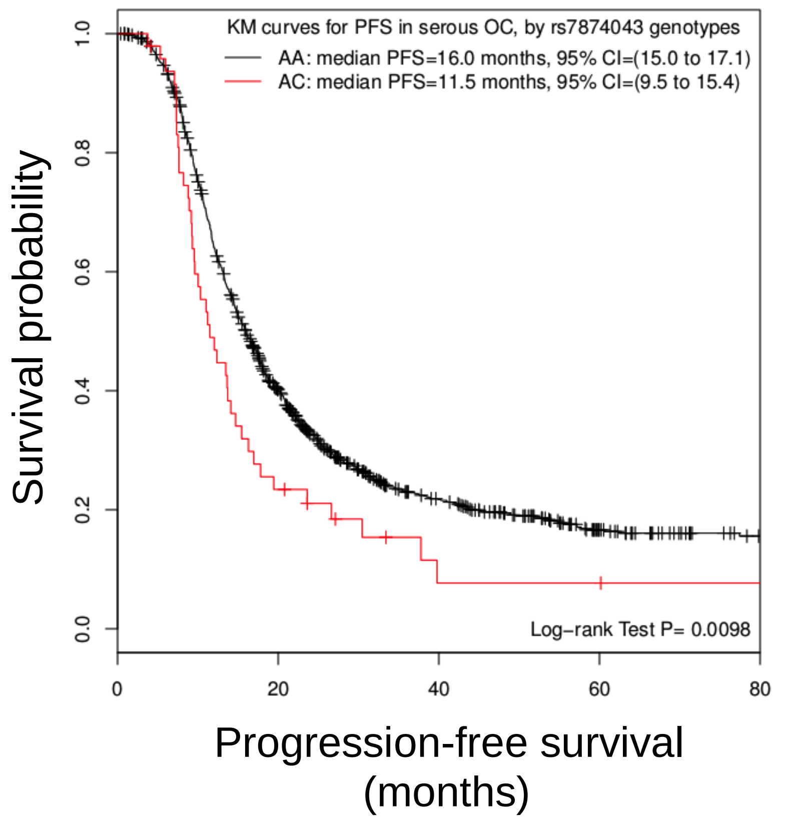

Supplementary Figure 2. **Unadjusted Kaplan-Meier (KM) curve for PFS by rs7874043 genotypes.** The KM curves compared PFS in patients with AA genotypes and with AC genotypes. Patients with CC genotypes were not observed due to the low minor allele frequency of rs7874043. The significance of differences in the KM curves was determined using a Log-rank Test. The survival curves were truncated at 80 months as only few events occurred after that.

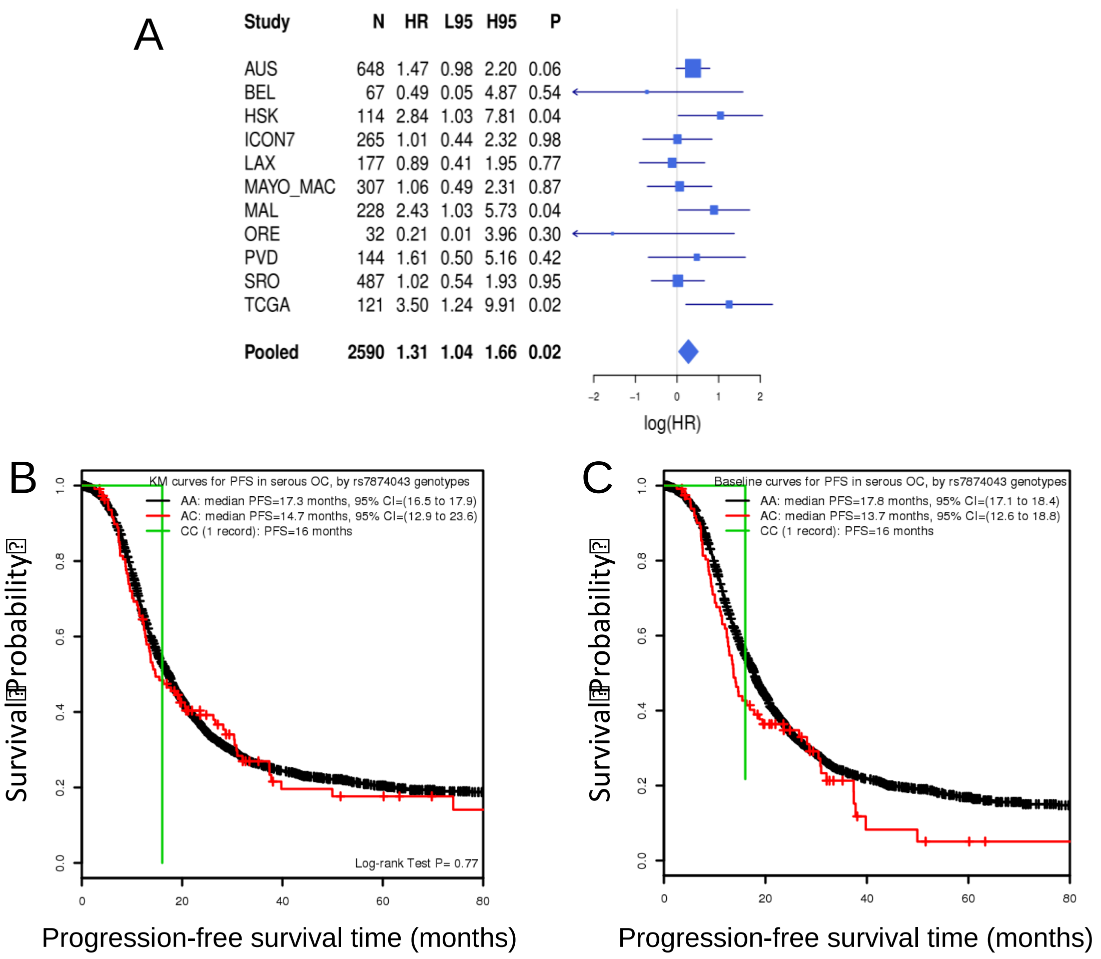

Supplementary Figure 3. Association with PFS in serous epithelial ovarian cancer (EOC) patients without restriction on chemotherapy. (A) Associations between the *TTC39B* SNP rs7874043 and PFS in individual studies and the overall association pooling all samples together while stratifying for studies. "HR" indicates the point estimates of hazard ratio. "L95" and "H95" represents its lower and upper 95% confidence intervals. The forest plot on the right is on the log scale. The arrow indicates that the estimate exceeds the scale of x-axis. **(B)** Unadjusted Kaplan-Meier (KM) curves and **(C)** baseline survival curves, of the three genotypes of rs7874043. The survival curves were truncated at 80 months as only few events occurred after that.

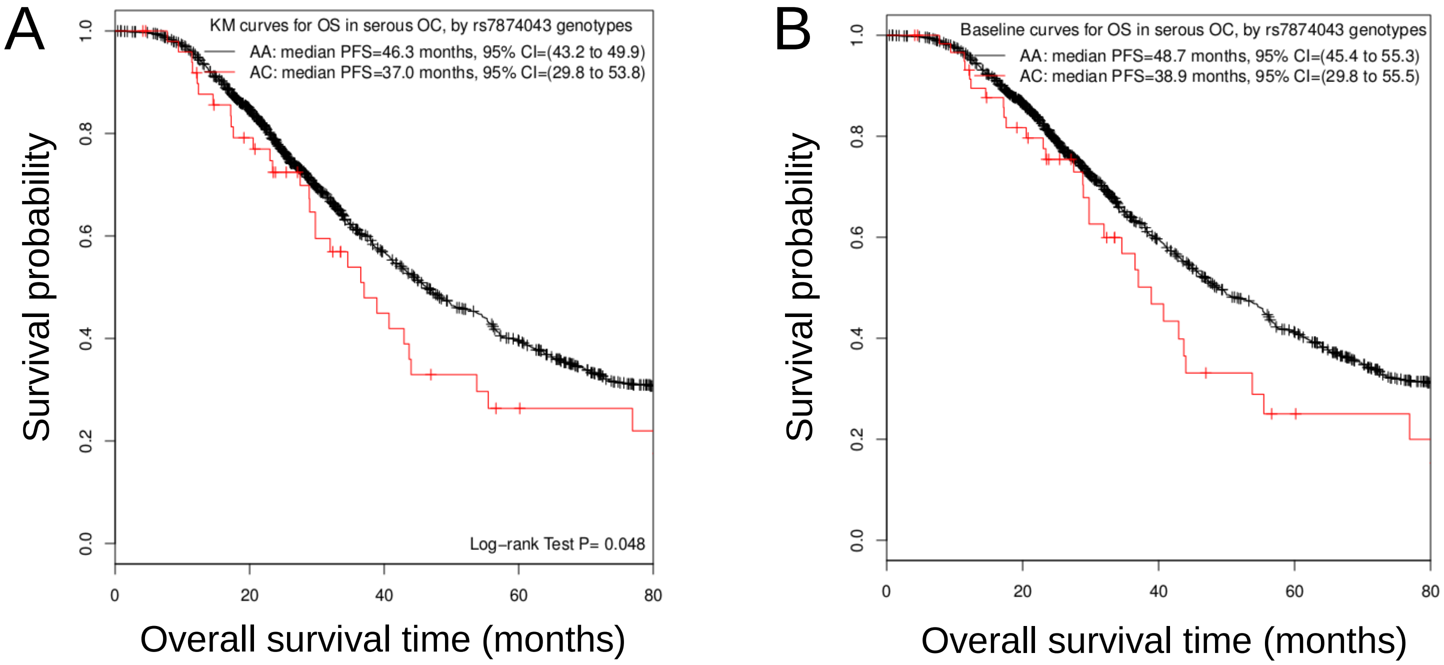

Supplementary Figure 4. **Unadjusted Kaplan-Meier curve (A) and baseline survival curves (B) for overall survival (OS) by rs7874043 genotypes.** The survival curves compared overall survival in patients with AA genotypes and with AC genotypes. Patients with CC genotypes were not observed due to the low minor allele frequency of rs7874043. The survival curves were truncated at 80 months as only few events occurred after that.

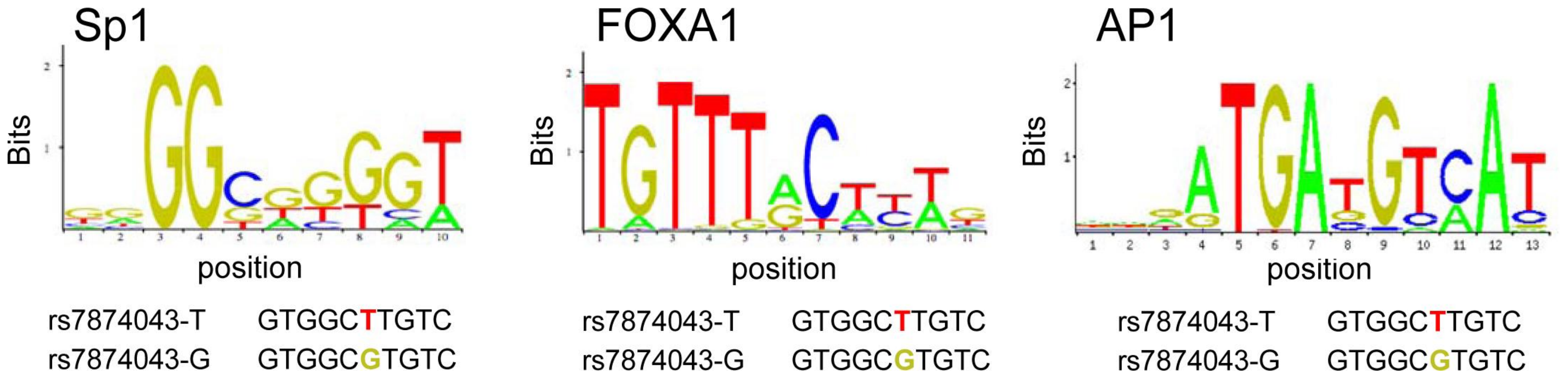

Supplementary Figure 5. **Transcription factor binding predictions. (A)** Position weight matrix (PWM) of Sp1, FOXA1 and AP1 from JASPAR, with homology to the PFS-associated (*g*) allele of rs7874043 colored below**.**

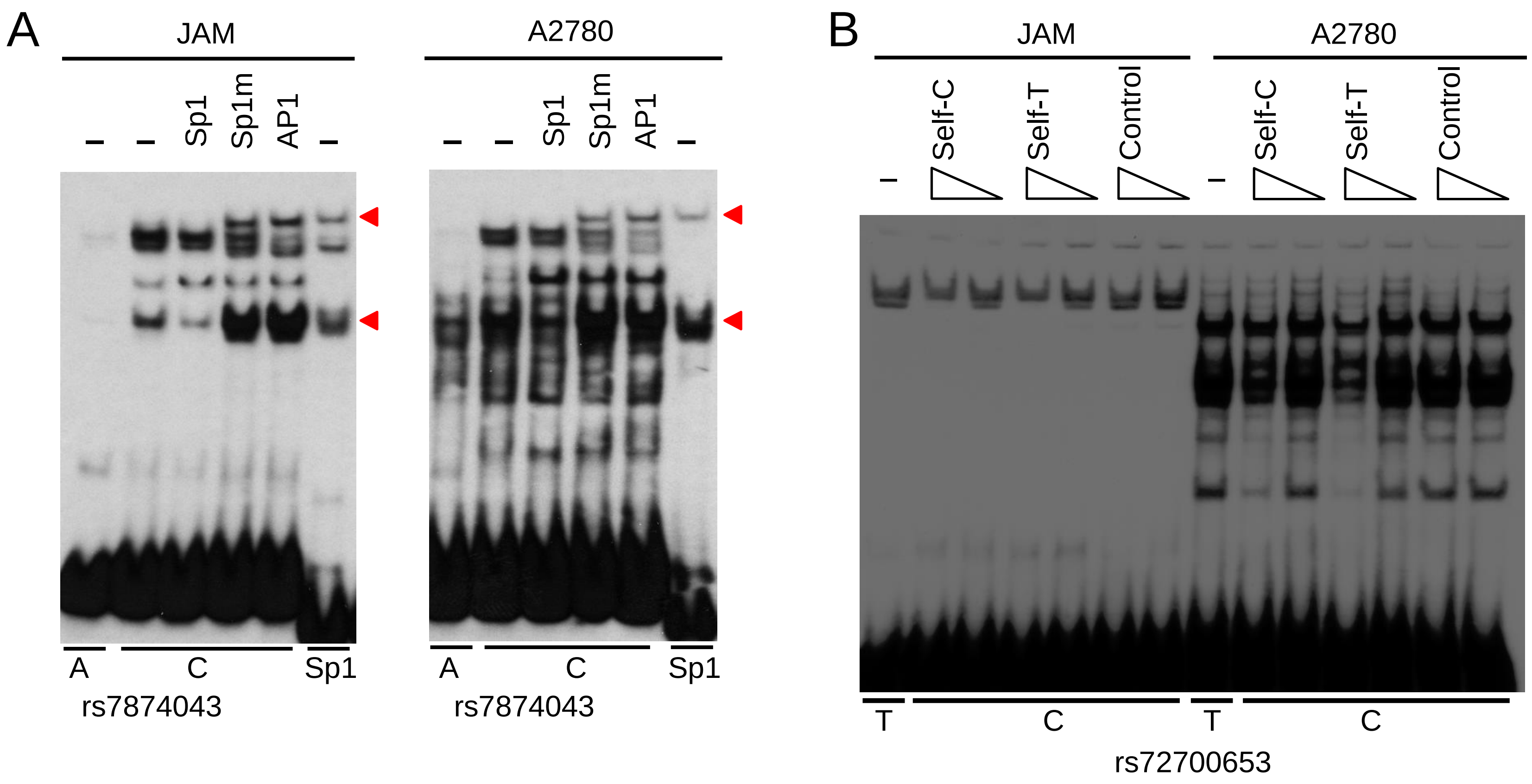

Supplementary Figure 6. **DNA-protein interactions surrounding the 9p22 PFS-associated SNPs. (A)** EMSAs using JAM or A2780 nuclear extract as per Figure 3b, except that additional controls were included. Competitor oligonucleotides are listed above each panel: (-) no competitor; Sp1, oligonucleotide with consensus Sp1 binding site; Sp1m, an identical oligonucleotide in which the Sp1 site itself was mutated; and AP1. In addition a radiolabeled Sp1 control oligonucleotide was included that yielded bands of the same mobility as those competed off by the Sp1 oligonucleotide (highlighted by the red arrow heads). The identity of the radiolabelled oligos is indicated below each panel. **(B)** EMSA for SNP rs72700653 (T=common allele, C=minor allele) in JAM and A2780 nuclear extracts**.** Labels above each lane indicate inclusion of competitor oligonucleotides at 30- and 10-fold molar excess, respectively. Control denotes a non-specific competitor (containing binding site for ATF, a TF not predicted to bind).

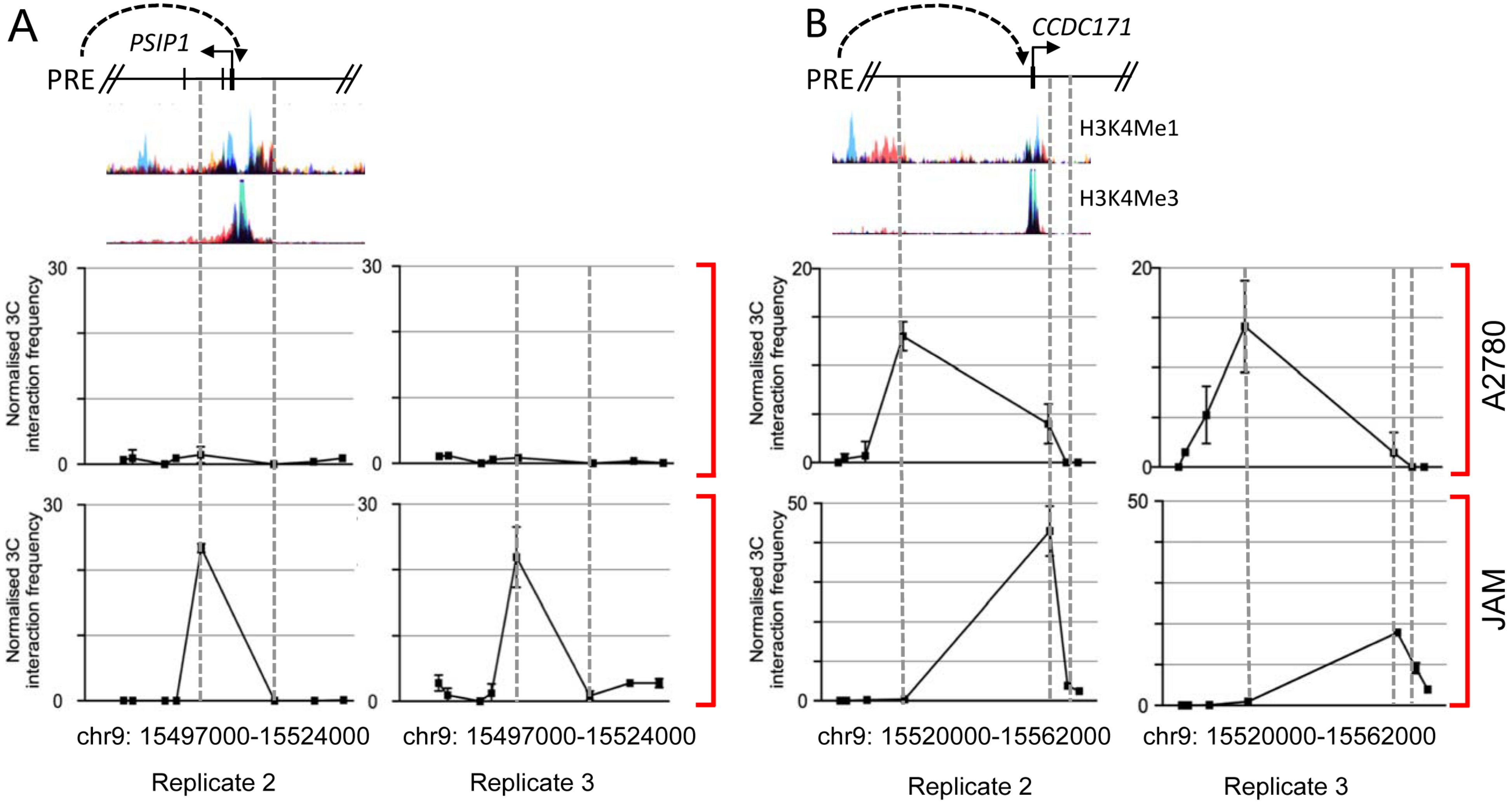

Supplementary Figure 7. **Chromatin interactions at 9p22 in ovarian cancer cell lines**. Replicate 3C interaction profiles between the putative regulatory element (PRE; containing rs72700653 and rs7874043) and **(A)** *PSIP1* or **(B)** *CCDC171* promoter regions. 3C libraries were generated with *Eco*RI, with the anchor point set at the PRE. A physical map of the region interrogated by 3C and relevant ENCODE histone modification data is shown above. Error bars denote SD.

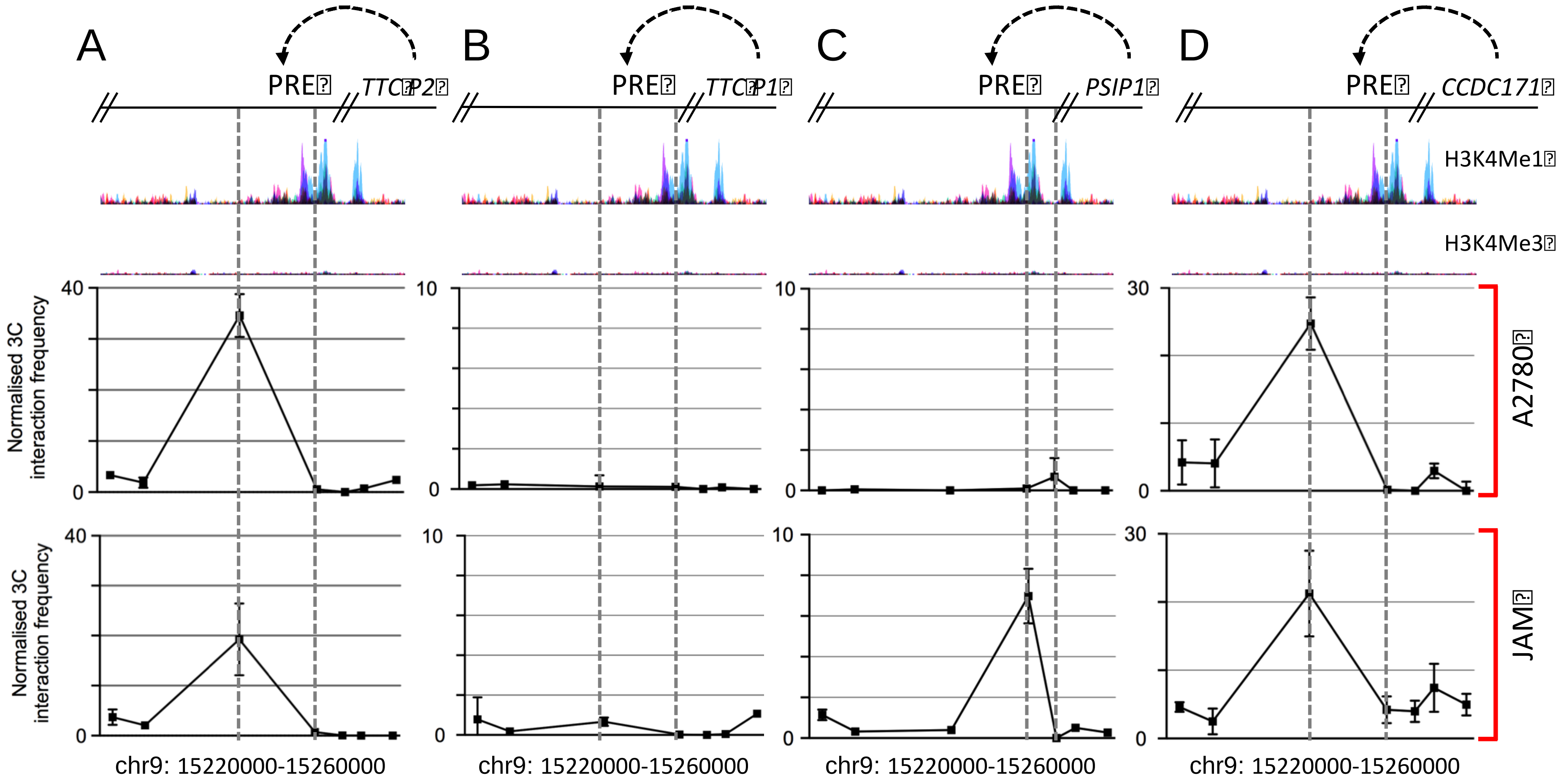

Supplementary Figure 8. **Chromatin interactions at 9p22 in ovarian cancer cell lines**. 3C interaction profiles between the putative regulatory element (PRE; containing rs72700653 and rs7874043) and **(A)** *TTC39B* alternative (P2), **(B)** TTC39B canonical (P1), **(C)** *PSIP1* or **(D)** *CCDC171* promoter regions. 3C libraries were generated with *Bgl*II, with the anchor point set at the relevant promoter. A physical map of the region interrogated by 3C and relevant ENCODE histone modification data is shown above. Error bars denote SD.

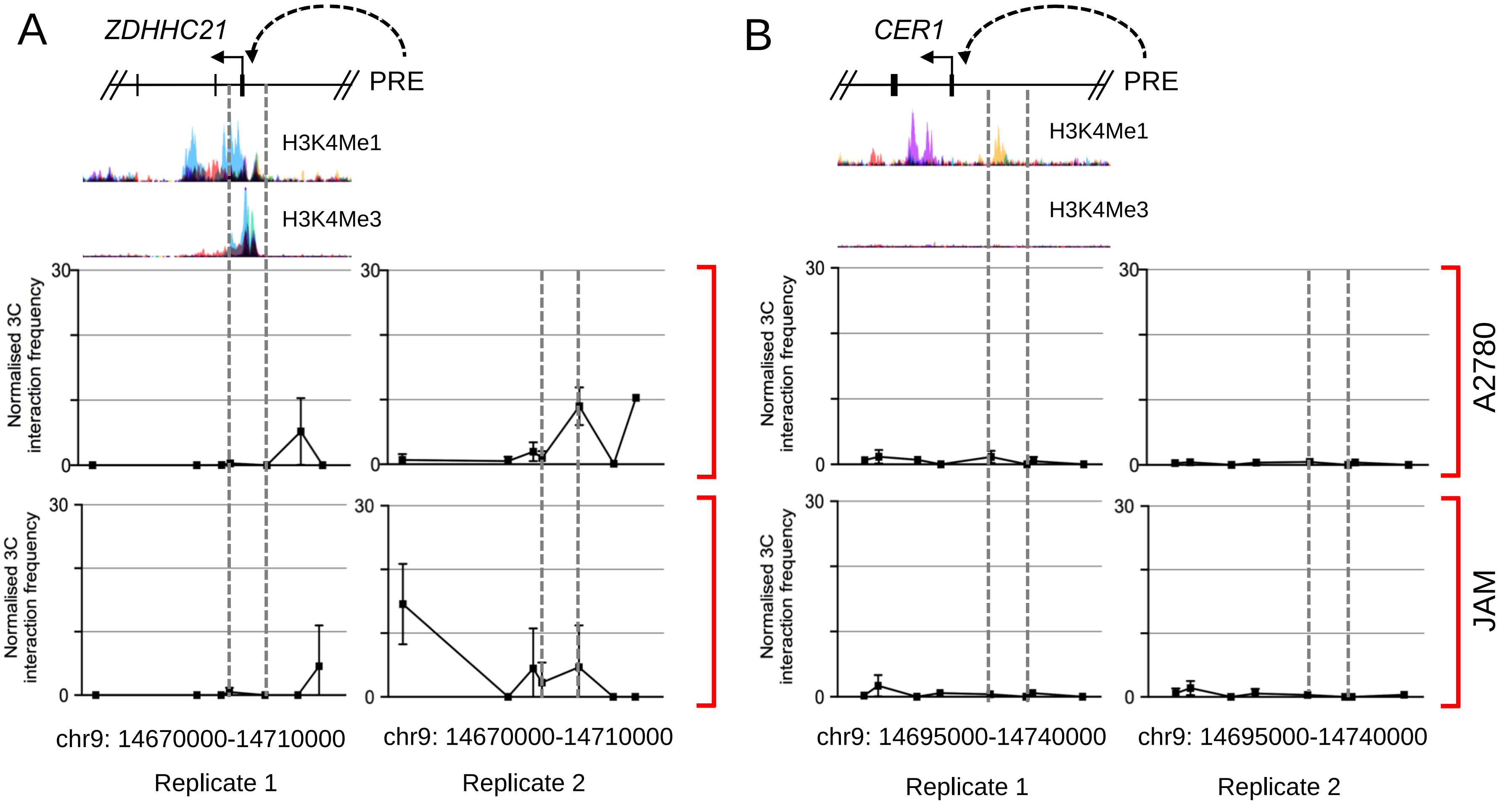

A
ZDHHC21
PRE
H3K4Me1
H3K4Me3
Normalised 3C interaction frequency
30
0
chr9: 14670000-14710000
chr9: 14670000-14710000
Replicate 1
Replicate 2
B
CER1
PRE
H3K4Me1
H3K4Me3
chr9: 14695000-14740000
chr9: 14695000-14740000
Replicate 1
Replicate 2
A2780
JAM

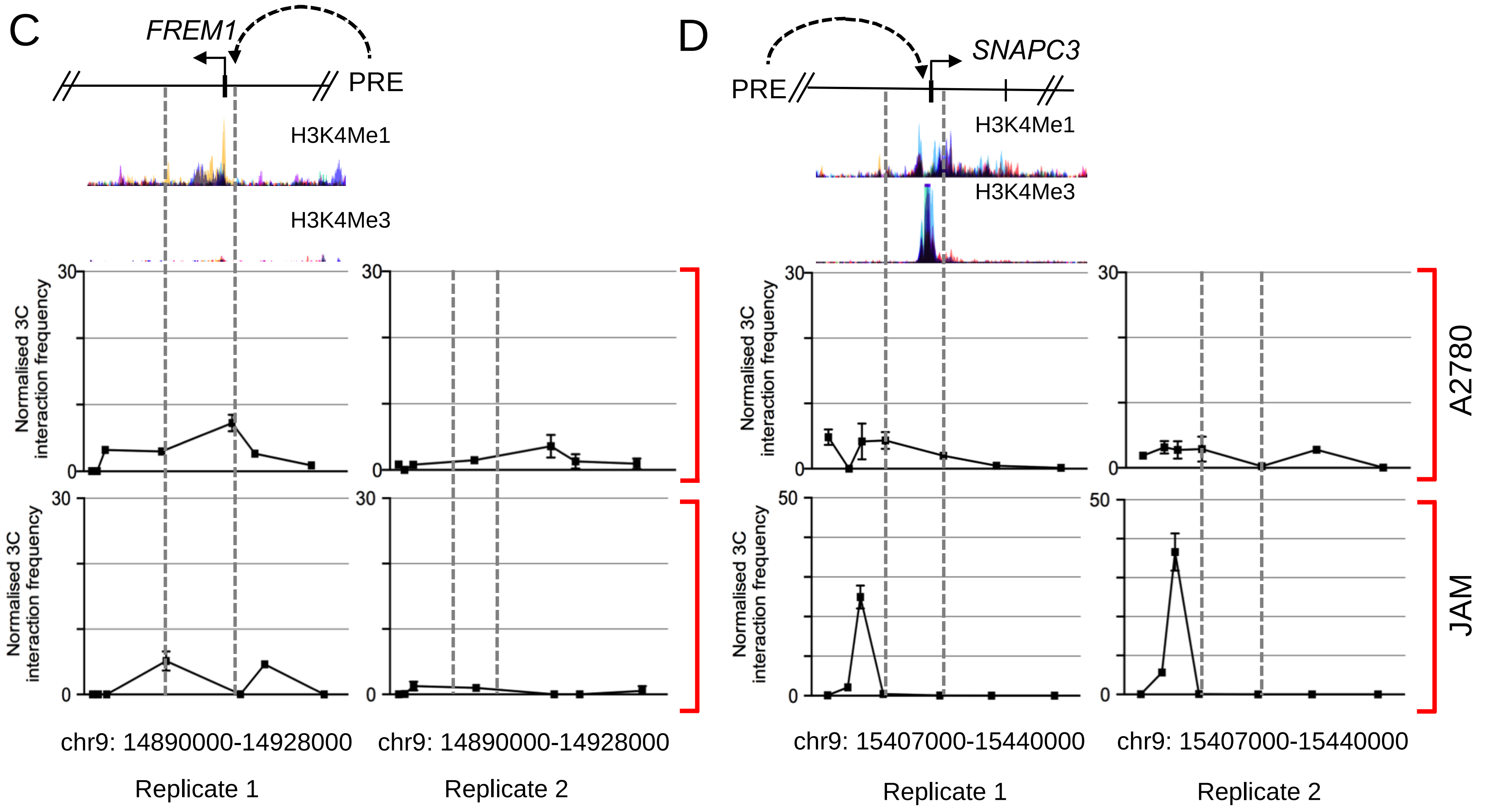

Supplementary Figure 9. **Chromatin interactions at 9p22 in ovarian cancer cell lines**. 3C interaction profiles between the putative regulatory element (PRE; containing rs72700653 and rs7874043) and **(A)** *ZDHHC21,* **(B)** *CER1,* **(C)** *FREM1* and **(D)** *SNAPC3* promoter regions. 3C was also performed with the *NFIB* promoter region but no interactions were detected. 3C libraries were generated with *Eco*RI, with the anchor point set at the PRE. A physical map of the region interrogated by 3C and relevant ENCODE histone modification data is shown above. Error bars denote SD.

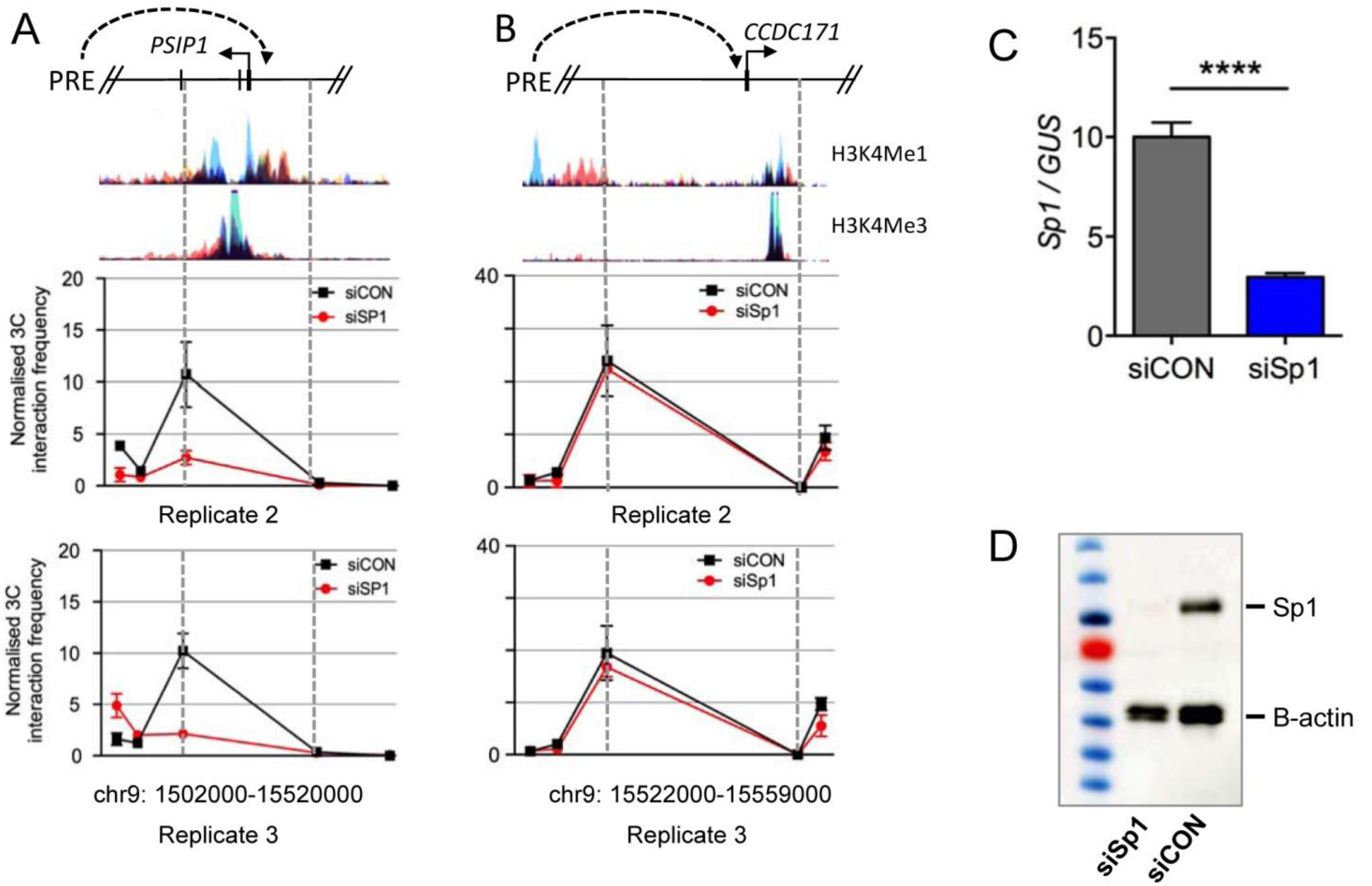

Supplementary Figure 10. **Chromatin interactions in JAM ovarian cancer cell lines following Sp1 siRNA treatment**. Replicate 3C interaction profiles between the putative regulatory element (PRE; containing rs72700653 and rs7874043) and **(A)** *PSIP1* or **(B)** *CCDC171* promoter regions. 3C libraries were generated with *Eco*RI, with the anchor point set at the PRE. A physical map of the region interrogated by 3C and relevant ENCODE histone modification data is shown above. Error bars denote SD. Sp1 RNA **(C)** and protein **(D)** levels in JAM cells following transfection of nontargeting control (siCON) or Sp1 (siSp1) RNAi smartpools. *B-glucuronidase* (GUS) or B-actin was used as loading controls, respectively. Error bars denote SEM (N=3). *P* values were determined with a two-tailed t test. ****p<0.0001.

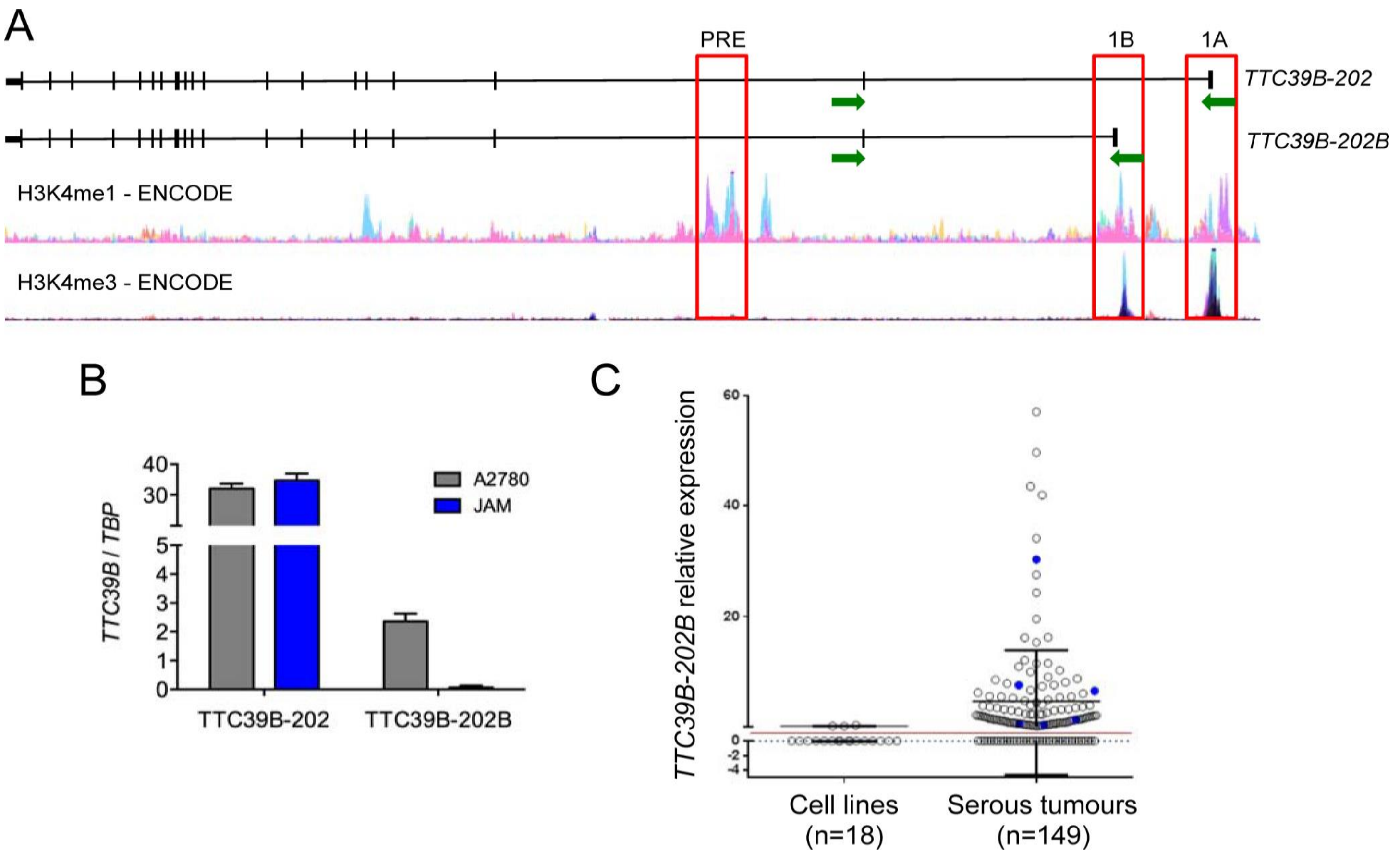

Supplementary Figure 11. **Identification and expression of a novel *TTC39B* transcript in ovarian cancer cell lines and tissues**. **(A)** Schematic of *TTC39B-202* (UCSC) and *TTC39B*-202B transcript identified through 5' and 3' RACE in this study. Colored histograms denote histone modification ChIP-seq data from ENCODE. Red boxes show the putative regulatory element (PRE) containing SNPs rs72700653 and rs7874043, canonical *TTC39B* promoter (1A) and novel *TTC39B* promoter (1B). Green arrows show primers used for RACE and qRTPCR **(B)** qRTPCR assay measuring *TTC39B* mRNA levels relative to *TATA-binding protein* (TBP) mRNA levels in A2780 and JAM cell lines. Graphs represent three independent experiments. Error bars denote SEM. **(C)** Relative expression of *TTC39B*-202B transcript in ovarian cancer cell lines (n=18) and serous ovarian epithelial tumors (n=149) was calculated by the ΔΔCt method. Samples were calibrated using the median ΔCt for the cohort. The red line denotes the level below which samples were non-quantitative or undetected. Blue circles indicate tumours heterozygous for rs7874043.

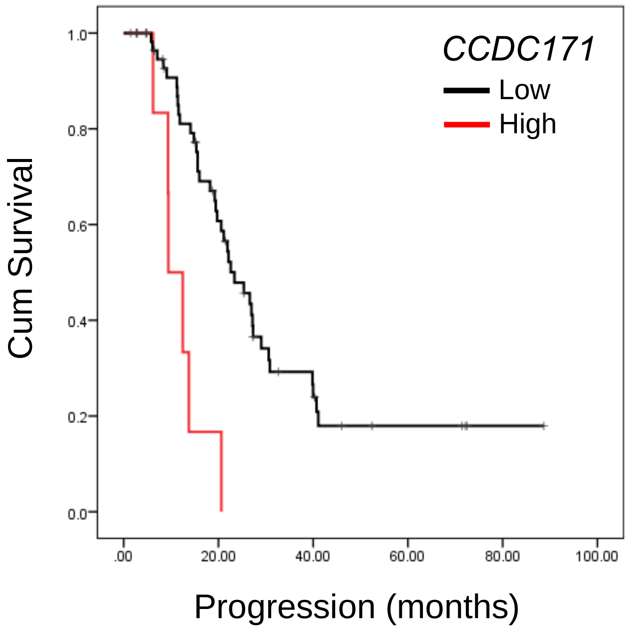

Supplementary Figure 12. Kaplan-Meier curves of association between expression of *CCDC171* with overall survival in epithelial ovarian cancer. Cum = cumulative. Expression of *CCDC171* for the upper decile vs remaining patients (log rank P = 0.001) with progression-free survival in 68 patients with serous epithelial ovarian cancer (selected for nil residual disease) in TCGA.

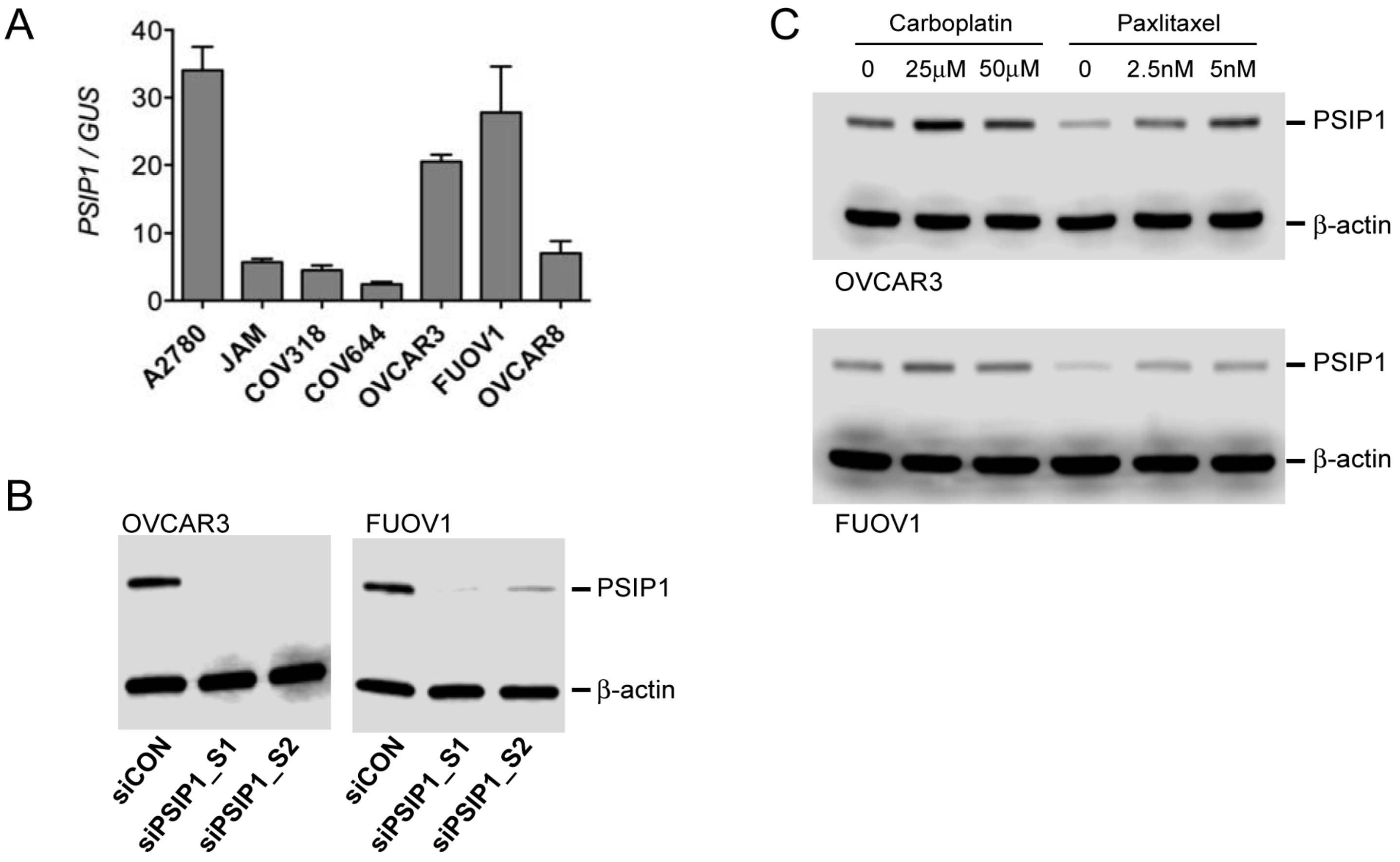

Supplementary Figure 13. **PSIP1 levels in ovarian cancer cell lines. (A)** *PSIP1* RNA levels in ovarian cancer cells. *B-glucuronidase* (GUS) was used as a loading control. **(B)** PSIP1 protein levels in ovarian cancer cells following transfection of nontargeting control (siCON) or two independent *PSIP1* (siPSIP1) siRNAs. β-actin was used as a loading control. **(C)** FUOV1 and OVCAR3 cells were seeded into 6-well plates at 7.5 x 10^5 cells per well. Cells were treated 16 h later with 25 µM and 50 µM of carboplatin (PBS as vehicle control), or 2.5 nM and 5 nM of paclitaxel (DMSO as vehicle control), incubated for a further 48 h, then protein extracted for Western blot analysis. β-actin was used as loading control.

Supplementary Table 1. Associations of 190 SNPs selected from Phase1 GWAS for replication, and 10 SNP in *YAP1*.

SNP	Chr	BP_b37	Gene	P_Phase 1[a]	P_Phase 2[b]	P_pooled[c]	SNP	Chr	BP_b37	Gene	P_Phase 1[a]	P_Phase 2[b]	P_pooled[c]
chr1:2921571	1	2921571	--	2.8E-05	0.12	0.031	rs2390487	2	167660965	--	7.7E-03	0.7	0.25
rs11209363	1	41485077	SLFNL1	2.3E-04	0.0017	0.066	rs17400325	2	178565913	PDE11A	6.3E-06	0.36	0.067
rs2236129	1	41617914	SCMH1	6.7E-04	0.018	0.17	rs73034264	2	178669674	PDE11A	5.6E-06	0.35	0.28
chr1:55051230	1	55051230	ACOT11	1.4E-06	0.67	0.01	rs73034271	2	178671155	PDE11A	7.9E-06	0.24	0.6
rs207145	1	55808143	--	7.4E-05	0.58	0.29	rs73034292	2	178679243	PDE11A	3.3E-05	0.33	0.42
chr1:76501317	1	76501317	--	4.7E-03	0.13	0.57	rs3821009	2	178682471	PDE11A	5.6E-06	0.58	0.022
rs12073224	1	92200963	TGFBR3	6.3E-06	0.29	0.029	rs1025598	2	178921642	PDE11A	1.5E-05	0.29	0.0089
rs12035421	1	96361709	--	2.7E-04	0.83	0.69	chr2:179013814	2	179013814	--	5.0E-05	0.13	0.66
rs61789271	1	96474723	--	3.8E-05	0.44	0.69	rs72911330	2	191002884	C2orf88	2.5E-02	0.3	0.041
rs11166552	1	101661978	--	7.1E-05	0.1	0.38	rs10221670	2	209040591	C2orf80	1.3E-01	0.1	0.074
rs790056	1	160969585	F11R	7.5E-05	0.97	0.22	rs28407435	3	13147337	--	1.5E-02	0.82	0.13
chr1:179923814	1	179923814	--	3.0E-06	0.33	0.006	rs28879123	3	16266489	GALNTL2	1.4E-05	0.62	0.55
rs16855014	1	179925538	CEP350	2.2E-05	0.42	0.0075	rs35804331	3	21770573	ZNF385D	2.9E-05	0.56	0.14
chr1:219133120	1	219133120	--	1.5E-07	0.41	0.085	rs62250946	3	43543023	ANO10	1.8E-05	0.54	0.14
chr1:231636516	1	231636516	--	6.8E-06	0.8	0.048	rs35225847	3	44299339	C3orf77	1.9E-04	0.75	0.16
chr1:236289189	1	236289189	--	2.4E-06	0.13	0.00074	rs1482622	3	83004712	--	2.1E-05	0.55	0.0086
rs12035655	1	236351833	GPR137B	9.9E-07	0.77	0.21	rs62258905	3	104375385	--	3.8E-05	0.35	0.25
rs811309	2	7869927	--	4.1E-06	0.16	0.66	rs13100275	3	162083007	--	3.3E-05	0.64	0.019
chr2:12141045	2	12141045	--	1.6E-06	0.8	0.12	rs34264209	3	162093889	--	3.6E-06	0.87	0.035
rs7604694	2	13681063	--	1.5E-03	0.4	0.0039	chr3:169735222	3	169735222	--	1.7E-05	0.37	0.47
rs11096718	2	17281115	--	8.7E-05	0.31	0.19	chr4:28644668	4	28644668	--	1.7E-05	0.43	0.061
rs62123793	2	17310155	--	6.4E-05	0.81	0.029	rs17830463	4	75218488	--	4.5E-02	0.0043	0.0037
rs62139377	2	41976649	--	3.5E-04	0.72	0.12	chr4:83590711	4	83590711	SCD5	2.7E-06	0.23	0.52
rs62134193	2	45054836	--	1.1E-05	0.63	0.032	rs72883270	4	94926423	--	3.4E-02	0.89	0.44
chr2:81741335	2	81741335	--	3.9E-04	0.27	0.61	rs3104107	4	141449275	ELMOD2	2.2E-05	0.18	0.76
chr2:104825558	2	104825558	--	1.9E-02	0.17	0.27	chr4:153135451	4	153135451	--	2.3E-07	0.26	0.056
rs10171182	2	144417583	ARHGAP15	2.6E-02	0.44	0.025	rs11940756	4	184296922	--	5.5E-06	0.43	0.047
rs72859654	2	144448659	ARHGAP15	2.9E-02	0.13	0.0017	chr5:10131285	5	10131285	--	2.7E-07	0.2	0.098
rs10497137	2	154691177	--	1.4E-05	0.27	0.019	rs9314069	5	164877178	--	6.7E-03	0.92	0.32
rs62174116	2	154717220	--	2.4E-05	0.82	0.17	rs12522758	5	164877481	--	1.5E-03	0.65	0.16
rs4339375	5	165652271	--	1.0E-07	0.6	0.53	rs36097937	9	14132910	NFIB	7.1E-05	0.93	0.19
rs1353920	5	165655853	--	3.1E-08	0.024	0.63	**rs72700653**	**9**	**15249050**	**TTC39B**	**7.8E-04**	**0.00019**	**3.5E-07**

chr5:168516390	5	168516390	SLIT3	1.2E-05	0.38	0.81	**rs7874043**	**9**	**15249431**	**TTC39B**	**7.9E-04**	**0.000069**	**3.6E-07**
rs7759455	6	2032887	GMDS	6.3E-03	0.24	0.051	rs55791531	9	15399766	--	3.9E-01	0.55	0.77
rs1539422	6	15950027	--	2.8E-05	0.47	0.036	rs10963675	9	18619044	ADAMTSL1	6.3E-05	0.11	0.00025
chr6:22950908	6	22950908	--	3.1E-05	0.81	0.39	rs10963676	9	18622043	ADAMTSL1	4.4E-05	0.66	0.11
chr6:22968035	6	22968035	--	1.1E-04	0.43	0.51	rs7865992	9	27189732	TEK	4.4E-03	0.76	0.39
rs62389569	6	22979636	--	5.8E-04	0.84	0.072	rs11144555	9	78264091	MIR548H3	1.2E-05	0.42	0.17
rs7754089	6	23115046	--	1.3E-05	0.21	0.26	rs1331924	9	107547622	ABCA1	4.3E-03	0.81	0.11
rs62399101	6	23180995	--	1.1E-04	0.41	0.51	rs10759126	9	108432696	--	2.4E-06	0.21	0.035
chr6:23193131	6	23193131	--	3.1E-03	0.57	0.8	rs7861233	9	117755116	--	2.2E-05	0.41	0.17
rs4712745	6	23193810	--	1.9E-03	0.84	0.13	rs7089727	10	1722488	ADARB2	2.1E-03	0.38	0.51
rs12195168	6	23197905	--	1.9E-03	0.55	0.22	rs73579126	10	9431550	--	1.6E-05	0.097	0.00064
rs62399106	6	23199025	--	1.6E-03	0.53	0.6	rs10827631	10	36648566	--	5.6E-04	0.38	0.74
rs61559613	6	74524178	CD109	1.8E-03	0.37	0.041	rs4148398	10	101592622	ABCC2	3.4E-01	0.65	0.89
chr6:74641387	6	74641387	--	3.4E-03	0.36	0.15	chr10:124411392	10	124411392	--	1.6E-02	0.64	0.2
chr6:77488849	6	77488849	--	1.2E-05	0.16	0.4	rs6483541	11	18866999	--	1.0E-04	0.68	0.0088
chr6:77815380	6	77815380	--	1.8E-05	0.37	0.71	rs584726	11	30095422	--	5.3E-06	0.34	0.4
rs6570115	6	137253162	--	5.1E-05	0.47	0.11	rs4755777	11	44124318	EXT2	1.9E-02	0.27	0.0004
rs9371821	6	154981953	--	6.5E-05	0.88	0.1	rs1820453	11	101980335	YAP1	7.2E-01	0.77	0.72
rs6462134	7	29423523	CHN2	1.1E-03	0.52	0.49	rs10895257	11	101986644	YAP1	5.2E-01	0.75	0.65
rs1362360	7	29506756	CHN2	1.1E-01	0.25	0.12	rs12420608	11	101992349	YAP1	1.7E-04	0.37	0.47
chr8:3633487	8	3633487	CSMD1	2.1E-06	0.093	0.39	rs11225138	11	101993898	YAP1	7.2E-01	0.23	0.58
rs2732987	8	5542087	--	2.2E-04	0.96	0.078	chr11:101539568	11	102034358	YAP1	5.1E-05	0.78	0.17
rs4875190	8	5549469	--	3.4E-05	0.9	0.04	rs10895269	11	102039296	YAP1	9.8E-01	0.088	0.071
rs72649453	8	59731018	TOX	5.6E-04	0.051	0.0058	rs1942683	11	102044647	YAP1	8.6E-01	0.21	0.069
chr8:68727023	8	68727023	--	1.4E-02	0.72	0.29	rs11602707	11	102045643	YAP1	9.2E-01	0.82	0.35
rs28512278	8	104288012	--	4.3E-02	0.53	0.13	rs17097475	11	102053823	YAP1	7.9E-01	0.44	0.94
rs1434520	8	108999623	RSPO2	3.1E-05	0.19	0.14	rs17689778	11	102063437	YAP1	7.1E-01	0.25	0.45
rs17789043	8	120342332	--	6.7E-06	0.24	0.31	rs11824640	11	134348650	--	1.7E-05	0.41	0.99
rs11780467	8	138199228	--	6.3E-05	0.13	0.9	rs73036394	11	134398777	--	6.5E-05	0.77	0.15
rs10967320	9	2644002	VLDLR	1.7E-09	0.13	0.52	rs10492110	12	9493492	--	9.3E-04	0.7	0.038
rs1929429	9	12480405	--	3.8E-06	0.55	0.52	rs61913576	12	9632555	--	8.1E-03	0.36	0.13
chr12:44529683	12	44529683	TMEM117	1.8E-05	0.84	0.15	chr16:88798398	16	88798398	FAM38A	1.4E-04	0.56	0.082
rs11170327	12	53307026	--	6.5E-06	0.2	0.36	chr16:88816894	16	88816894	FAM38A	1.2E-03	0.27	0.055
rs1861932	12	68886533	--	1.4E-02	0.37	0.032	rs11076712	16	88820353	FAM38A	1.1E-02	0.21	0.14

rs3860254	12	80804476	--	3.8E-05	0.14	0.0016	chr17:752428	17	752428	NXN	1.5E-05	0.26	0.39
rs17193501	12	91172990	--	8.4E-06	0.88	0.013	rs8066936	17	754600	NXN	1.9E-06	0.91	0.092
rs12308739	12	91187331	--	2.6E-05	0.47	0.0036	rs56803883	17	755097	NXN	1.6E-06	0.84	0.12
rs11105855	12	91274561	--	3.0E-06	0.35	0.0043	rs12449568	17	54430155	ANKFN1	3.4E-04	0.57	0.017
rs17021407	12	94013808	--	1.3E-07	0.64	0.67	rs7212686	17	66923711	ABCA8	5.8E-03	0.45	0.1
rs257963	12	97710988	--	6.8E-04	0.27	0.9	rs6502005	17	67103511	ABCA6	6.0E-01	0.56	0.74
chr12:97717747	12	97717747	--	2.3E-04	0.8	0.016	rs536009	17	67273882	ABCA5	1.2E-03	0.012	0.00031
rs71460383	12	97720887	--	1.7E-03	0.35	0.029	chr17:75162047	17	75162047	SEC14L1	1.5E-05	0.57	0.12
rs1878022	12	108699032	CMKLR1	4.3E-01	0.46	0.96	rs7214455	17	75599604	--	6.1E-05	0.91	0.088
rs17075983	13	31991953	--	4.2E-05	0.64	0.19	rs55682777	18	45511144	--	7.2E-06	0.7	0.29
rs2314972	13	31992393	--	4.6E-03	0.22	0.057	rs1011420	18	46282982	KIAA0427	5.4E-03	0.89	0.13
rs12584266	13	31994485	--	2.0E-03	0.64	0.053	rs6566916	18	55674257	--	4.5E-05	0.21	0.017
rs716276	13	32012208	--	3.9E-04	0.52	0.32	rs1942528	18	55676499	--	1.7E-06	0.4	0.3
rs7989641	13	64126078	--	1.7E-06	0.029	0.0014	rs72939535	18	63620142	--	4.6E-05	0.095	0.0032
rs9600222	13	74652873	KLF12	7.3E-05	0.69	0.12	rs4510132	18	64021299	--	6.0E-05	0.21	0.0044
rs9566037	13	85728432	--	3.5E-05	0.034	0.55	rs12458066	18	64252555	CDH19	9.4E-05	0.82	0.1
rs9602695	13	85807556	--	2.5E-05	0.3	0.41	rs73542498	19	30100002	POP4	3.5E-06	0.86	0.78
rs7335794	13	85838725	--	4.8E-05	0.44	0.42	rs73057016	19	53826943	--	1.3E-03	0.59	0.0073
rs12431174	13	85840960	--	3.0E-06	0.8	0.004	chr20:40563880	20	40563880	--	3.0E-05	0.069	0.00077
rs12879346	14	23652708	SLC7A8	7.4E-05	0.34	0.76	rs55699935	20	55361541	--	2.0E-02	0.24	0.98
rs880979	14	31056351	G2E3	9.6E-06	0.58	0.17	rs59635584	20	55364044	--	1.7E-02	0.21	0.75
rs12589770	14	41130279	--	1.6E-02	0.72	0.44	rs1048672	21	35210184	ITSN1	1.1E-07	0.89	0.25
chr15:44543579	15	44543579	--	2.1E-05	0.32	0.6	chr21:37781046	21	37781046	CHAF1B	3.1E-05	0.79	0.48
rs893912	15	66928469	--	3.2E-06	1	0.01	rs2267347	22	37012953	CACNG2	2.7E-05	0.83	0.043
rs8023562	15	66941774	--	4.8E-05	0.56	0.039	rs73166104	22	43705158	SCUBE1	2.8E-05	0.038	0.0081
rs7169032	15	67259899	--	5.0E-02	0.93	0.6	rs2858527	22	49716886	--	2.0E-05	0.47	0.017
chr16:52730910	16	52730910	--	8.8E-04	0.24	0.011	rs848732	22	49737239	--	1.8E-06	0.39	0.046
rs2398144	16	56352854	GNAO1	3.0E-05	0.48	0.25	rs708444	22	49740513	--	3.1E-04	0.25	0.0026
rs1061238	16	88781850	FAM38A	1.3E-04	0.28	0.05							

[a] P-values from Phase 1 GWAS (N=385). [b] P-values from Phase 2 replication in OCAC (N=706). [c] P-values from pooled Phase 1 and 2, removing ineligible samples from Phase 1 (N=985).

Supplementary Table 2. Chromosome 9 *TTC39B* fine-mapping SNPs

SNP	BP	MAF	$R^{2,a}$	P_Phase 1	P_Phase 2	P_pooled	SNP	BP	MAF	$R^{2,a}$	P_Phase 1	P_Phase 2	P_pooled
rs7874043	15249431	0.02	1	7.89E-04	0.000069	3.60E-07	rs12684860	15210451	0.2	0.015	0.922	0.2	0.22
rs72700653	15249050	0.02	0.896	7.83E-04	0.00019	3.50E-07	rs10961939	15256956	0.14	0.012	0.783	0.54	0.39
rs7023109	15268991	0.04	0.456	0.134	0.00017	1.20E-04	rs7875255	15290174	0.14	0.012	0.890	0.27	0.17
rs10122202	15279848	0.04	0.378	0.107	0.003	1.30E-03	rs562177	15283433	0.23	0.01	0.427	0.46	0.62
rs80137366	15282029	0.03	0.331	0.106	0.0021	1.40E-03	rs569078	15205014	0.34	0.008	0.677	0.4	0.56
rs10961955	15284640	0.04	0.279	0.007	0.074	1.60E-03	rs10961916	15212828	0.4	0.006	0.899	0.29	0.64
rs12003180	15283097	0.04	0.251	0.014	0.063	1.20E-03	rs10810360	15188743	0.3	0.005	0.488	0.0093	0.01
rs7037559	15282851	0.05	0.251	0.015	0.0045	1.50E-03	rs693196	15202417	0.26	0.005	0.328	0.016	0.01
rs12345299	15247714	0.09	0.171	0.321	0.048	2.50E-02	rs10961949	15276926	0.07	0.004	0.192	0.084	0.06
rs72700650	15248864	0.08	0.137	0.723	0.076	6.10E-02	rs12341152	15235161	0.06	0.004	0.459	1	0.77
rs10120504	15277905	0.1	0.1	0.040	0.00091	4.00E-04	rs11791131	15216292	0.12	0.004	0.590	0.75	0.67
rs471364	15289578	0.11	0.089	0.801	0.28	0.15	rs583204	15299715	0.1	0.003	0.435	0.15	0.89
rs3933784	15287247	0.11	0.089	0.824	0.2	0.26	rs10961934	15248194	0.09	0.002	0.983	0.53	0.75
rs4237138	15286386	0.11	0.087	0.828	0.25	0.14	rs7467520	15201034	0.39	0.002	0.158	0.14	0.06
rs10756655	15250501	0.5	0.07	0.046	0.99	0.81	rs619105	15240454	0.49	0.002	0.507	0.87	0.84
rs10810373	15293620	0.36	0.039	0.088	0.4	0.37	rs13300482	15260948	0.1	0.002	0.942	0.46	0.84
rs612183	15282158	0.23	0.033	0.975	0.18	0.2	rs552693	15267981	0.34	0.002	0.079	0.016	0.04
rs10810364	15218408	0.29	0.022	0.223	0.23	0.15	rs1575469	15180751	0.24	0.001	0.205	0.88	0.46
rs10810367	15270247	0.25	0.015	0.368	0.25	0.37	rs1407977	15188106	0.4	0.001	0.168	0.25	0.05
rs7024176	15191562	0.3	0.015	0.339	0.18	0.32	rs4087078	15297398	0.47	0.001	0.166	1	0.95

[a] R^2 indicates LD between the corresponding SNP with rs7874043.

Supplementary Table 3. Description, study design and patient ascertainment for all OCAC sites participating in the replication stage.

Site (Country)	Study Name	Design	Age Range	Sample size (restricted chemo/all chemo)	Design Exclusion Criteria	Ascertainment	Definition of Progression Free Survival
AOCS/ACS (as AUS, Australia)	Australian Ovarian Cancer Study/ Australian Cancer Study	Population-based	19-79	408/648	Previous ovarian cancer, non-epithelial ovarian cancer	Diagnosed from 2002 onwards; recruited through surgical treatment centers throughout Australia & cancer registries of Queensland S. Australia & W. Australia (AOCS) & cancer registries of New South Wales & Victoria (ACS)	The time interval between the date of histologic diagnosis and the first confirmed sign of disease recurrence, or progression, defined by the Gynaecologic Cancer InterGroup (GCIG), based on CA125 and incorporating RECIST, as previously described (1, 2)
BAV (Germany)	Bavarian Ovarian Cancer Cases and Controls	Hospital-based	19-88	57/73	Non-epithelial ovarian cancer	Cases were patients at the Gynecologic Oncology Center at the Comprehensive Cancer Center Erlangen-Nuremberg, Department of Gynecology and Obstetrics, Erlangen University Hospital.	Same as AOCS
BEL (Belgium)	Belgian Ovarian Cancer Study	Hospital-based	14-85	39/67	Unverified or non-epithelial ovarian cancer; cases unable to provide blood sample or too ill to participate; patients not able to read or speak Dutch, French or English and sign the consent	Cases attending the Gynecologic Oncology Unit at the Leuven University Hospital diagnosed with incident ovarian cancer from 2009 onwards	Same as AOCS

HSK (Germany)	Dr. Horst-Schmidt Kliniken, Wiesbaden	Hospital-based	17-92	77/114	Non- ovarian cancer	Cases attending the Gynecologic Oncology Unit at the Dr. Horst-Schmidt Kliniken, Wiesbaden, diagnosed with incident ovarian cancer between 2005-2009	Same as AOCS
The International Collaborative Ovarian Neoplasm study (ICON)7 trial	A randomised 2 arm, multi-centre, Gynecologic Cancer InterGroup (GCIG) open-label phase III trial designed to evaluate the safety and efficacy of adding bevacizumab, to standard chemotherapy (carboplatin and paclitaxel), in patients with advanced epithelial ovarian or primary peritoneal cancer.	Clinical trial	27-77	124/265	Patients with non-epithelial ovarian cancer, including malignant mixed Mullerian tumours, borderline tumours (tumours of low malignant potential), planned intraperitoneal cytotoxic chemotherapy, prior systemic anti-cancer therapy for ovarian cancer, surgery within 4 weeks prior to anticipated first dose of bevacizumab	Patients were enrolled between 2006 and 2009 at 263 centers in the United Kingdom, Germany, France, Canada, Australia,New Zealand, Denmark, Finland, Norway,Sweden, and Spain; and were randomly assigned in a 1:1 ratio to receive carboplatin-paclitaxel (standard-chemotherapy group), or to the same regimen plus bevacizumab	Disease progression was defined according to the Response Evaluation Criteria in Solid Tumors (RECIST) guidelines on the basis of radiologic, clinical, or symptomatic indicators of progression and did not include isolated asymptomatic progression on the basis of CA-125 levels (3).
LAX (USA)	Women’s Cancer Research Institute (Cedars-Sinai Medical Center)	Hospital-based	18 and above	87/177	Non-epithelial ovarian cancer	Cases are identified through the Women's Cancer Research Institute biorepository from 1989 onwards. Patients presenting to the gynecologic cancer service with epithelial ovarian	Same as AOCS

						cancer are identified in IRB 901 (Tissue Bank) and IRBs 1080 and 4049 (Gilda Radner Hereditary Cancer)	
MAL (Denmark)	The Danish Malignant Ovarian Tumor Study	Population-based	35-79	84/228	Unverified or non-epithelial ovarian cancer; cases unable to provide blood sample or too ill to participate	Incident cases diagnosed 1994 -1999 from municipalities of Copenhagen & Frederiksberg & surrounding counties.	The time interval was determined by ultrasonography, or defined by cancer antigen 125 (CA125) values (increase in CA125 to >35 U/mL from a CA125 value lower than 35 U/mL after the primary treatment)
MAYO/MAC (USA)	Mayo Clinic Ovarian Cancer Case Control Study	Clinic-based	>20	71/307	Previous ovarian cancer, non-epithelial ovarian cancer	Cases attending Mayo Clinic diagnosed from 2000 onwards identified in a six state surrounding region.	The time interval between the date of histologic diagnosis and progression, defined as radiographic evidence of recurrence or initiation of second-line therapy.
ORE (USA)	Oregon Ovarian Cancer Registry	Mixed	20-80	17/32	Prior history of ovarian cancer	Cases diagnosed from 2007, identified from the cancer registry, and a tissue repository of patients with ovarian cancer.	Same as AOCS
PVD (Denmark)	Pelvic Mass Study	Hospital-based	31-88	0/144	Unverified diagnosis of ovarian cancer or previous diagnosed ovarian cancer; cases unable to provide blood sample	All patients admitted with a pelvic mass at Rigshospitalet, University of Copenhagen, are included in the study with a blood sample less than 14 days before surgery/diagnosis of ovarian cancer and with FFPE and fresh frozen tissues if possible	Same as MAL
SCO (UK)	The Scottish Randomized Trial in Ovarian Cancer	Hospital-based/Clinical trial	18-84	216/487	Adenocarcinomas of unknown origin; Prior history of cancer within 5 years (except localized cervical or non-melanoma skin cancer) or concurrent	Patients randomized into a prospective phase III comparison of paclitaxel-carboplatin versus docetaxel-carboplatin as first line chemotherapy in stage Ic-IV epithelial ovarian cancer (SCOTROC	The time interval between the date of histologic diagnosis and progression, defined as (i) a 25% or greater increase in the size of at least 1 bi- or uni-dimensionally measurable lesion, (ii) a clear worsening from previous assessment of any evaluable

					malignancy; FIGO stages other than 1c to IV; Treatment with chemo- or radiotherapy prior to RCT; Advanced disease defined by inadequate bone marrow, renal or liver function or severe co-morbidities that contraindicate trial participation.	1) which recruited from 1998 to 2000	disease (note that worsening of existing non-evaluable disease did not constitute progression), (iii) the reappearance of any lesion that had disappeared, with the exception of ascitic or pleural fluid that was drained and recurred within 3 months of drainage, or (iv) the appearance of any new lesion and/or site.

Supplementary Table 4. Correlation between expression of *PSIP1* and other genes on the short arm of chr 9 in TCGA from cBioPortal.

Gene	Pearson Score	Spearman Score	Adjusted Score[1]
HAUS6	0.72	0.72	0.32
SNAPC3	0.67	0.68	0.21
CNTLN	0.66	0.65	0.60
NFIB	0.62	0.64	0.37
CCDC171	0.62	0.61	0.44
UHRF2	0.58	0.56	0.36
KIF24	0.56	0.58	0.51
DENND4C	0.56	0.56	0.17
FANCG	0.55	0.56	0.35
KIAA0020	0.55	0.55	0.40
MTAP	0.55	0.58	0.16
MPDZ	0.55	0.53	0.41
ZDHHC21	0.55	0.56	0.31
PLAA	0.54	0.55	0.21
RRAGA	0.54	0.55	0.05
DCAF12	0.53	0.52	0.36
UBE2R2	0.53	0.54	0.47
RFX3	0.53	0.52	0.52
IFT74	0.53	0.54	0.39
TOPORS	0.52	0.52	0.31
KLHL9	0.52	0.56	0.21
MELK	0.51	0.53	0.48
RCL1	0.51	0.51	0.14
KIF15	0.5	0.51	0.53
KIAA2026	0.5	0.49	0.40

[1] Correlation between residual expression levels after regressing out effects of copy number and methylation.

Supplementary Table 5. Treatment regimens uses for cases who did not have standard carboplatin/paclitaxel treatment

# cases	Treatment regimen
360	carboplatin IV AUC 5 + docetaxel IV 75 mg/m^2 every 3 weeks
233	paclitaxel and carboplatin - known or assumed standard doses but <4 cycles
215	minimum cytoreductive surgery; chemotherapy data not available or insufficient to assign regimen
141	standard paclitaxel/carboplatin + bevacizumab
133	monotherapy – carboplatin, paclitaxel or other drug
94	standard paclitaxel/carboplatin + other drug/regimen
71	carboplatin/paclitaxel and gemzar/topotecan/doxorubicin/cyclophosphamide/other drug
39	carboplatin + other drug
38	cisplatin and other drug
22	paclitaxel and carboplatin (non-standard doses)
**1346**	**TOTAL**

Supplementary Table 6. Oligonucleotides used in this study.

3C Primers	3C Fragment (hg19 coordinates)	Sequence (5' to 3')
TTC39B PRE bait	15,236,622	AATGGCAAAGAATCCCTTAAGCCAATGGG
TTC39B HindIII Fragment 1	15,255,800	GCAAATCCTAGCAGTCTACTCTGAGGATCCACC
TTC39B HindIII Fragment 2	15,256,991	GCGGAGAGAGAGGAAAGTGAACGGATGG
TTC39B HindIII Fragment 3	15,259,198	CAATTTATGGTTCGAAAGAGAAGGTGTGGAGAAGG
TTC39B HindIII Fragment 4	15,261,729	TGGAATCCTGAGGTGGTGTGAATACAGTGG
TTC39B HindIII Fragment 5	15,272,692	CCAAGGATGCAGGAAACTGTATTTGCTGG
TTC39B HindIII Fragment 6	15,276,192	TTCGGAAGAGGCAGTTCCCAAAGAGTAAGG
TTC39B HindIII Fragment 7	15,280,207	TCATACAGTGCCCTCCCTCTCTTGGTATCTATCC
TTC39B HindIII Fragment 8	15,282,448	ACCCTCTACAACACTCTATGAGCCAGACACTGC
TTC39B HindIII Fragment 9	15,283,778	CACATCTGGCAGTCACAACTCTTTAGCCTCC
TTC39B HindIII Fragment 10	15,287,301	CTGTGCCTCTGGATATAAATGCCCTTAGGAGG
TTC39B HindIII Fragment 11	15,288,954	CGGTCTTGATTTGTGACACCTAACACTTGTGC
TTC39B HindIII Fragment 12	15,291,295	AGTTGAACAAAAGGGACCGTCACTGGATCC
TTC39B HindIII Fragment 13	15,292,707	CCCTGAAGACTGTGACATTGGTGATCCTTACC
TTC39B HindIII Fragment 14	15,293,760	AAGCAGGCGGTAAAAACATGTCACATAAGCC
TTC39B HindIII Fragment 15	15,302,885	CCCAATGGTAGCCCATAAGTATGTGAAGGTAACC
TTC39B HindIII Fragment 16	15,305,022	AGGCTAGTACAGGGCTTGGCACCTAGTCAGC
TTC39B HindIII Fragment 17	15,305,963	TGGTAGGTGCCTTAGAGAAGGCATAGTTTTGCC
TTC39B HindIII Fragment 18	15,308,289	CATGAAGCCATCTCACTGGTAGCTTCTTCTCC
TTC39B HindIII Fragment 19	15,310,861	CATTTGACATCCGGGAAGGCTATTTGAAGG
TTC39B HindIII Fragment 20	15,316,772	GCAAATTGCTTGCCATGATTTAGAACAGTGG
TTC39B HindIII Fragment 21	15,318,137	TGTTCAAGGTCAGAAACCCAGGTCTCAAGG
PSIP1 EcoRI Fragment 1	15,498,333	CGAACTCAGACCTCAAATACCAATGTCAAAGC
PSIP1 EcoRI Fragment 2	15,499,347	GTACACACTTAGGGCTTCAACGTGTGTGCC
PSIP1 EcoRI Fragment 3	15,502,942	AGACCAACATACATATGGGGCAGCACTGC
PSIP1 EcoRI Fragment 4	15,504,215	CCACCTGTTTCCACTGTTGTTTCTTTGCAG
PSIP1 EcoRI Fragment 5	15,506,988	CCTCATGTGATTCAGGCACATCAGTGAAGG
PSIP1 EcoRI Fragment 6	15,515,153	CCTTCATCACGATTTTGGTGATGTCTGTTAGG
PSIP1 EcoRI Fragment 7	15,519,608	CCTGGATTACGATCACAGCAGAAAGCTCC
PSIP1 EcoRI Fragment 8	15,522,834	CTCTGGGTTAAATGGCCAGAAGTATCATTACTGG

CCDC171 EcoRI Fragment 1	15,522,834	CTCTGGGTTAAATGGCCAGAAGTATCATTACTGG
CCDC171 EcoRI Fragment 2	15,526,937	CCCTGATCGACAAAGACATTGGAACTTAATAACC
CCDC171 EcoRI Fragment 3	15,532,663	CACAGGTGTGGTTACTAATGAAGACAGCACTGC
CCDC171 EcoRI Fragment 4	15,554,784	CTGAGATCAGCATTCCTGATCTCATTTAATCCC
CCDC171 EcoRI Fragment 5	15,559,223	AGTTGCAAGGAACAGGGAGAAGTCAGTGG
CCDC171 EcoRI Fragment 6	15,564,215	CACAGACTTTTGTGGTCCAAATTAACAGAGGAGC
FREM1 EcoRI Fragment 1	14,891,207	TGAGGTCTTGTGGGAGGGAGATTTATAATCAGG
FREM1 EcoRI Fragment 2	14,892,041	GTTGGAAGCCTCTTCAGTATCATCCCTTTGG
FREM1 EcoRI Fragment 3	14,893,250	CCTAGGCCCGCATTCTATCCTGAGGTATCC
FREM1 EcoRI Fragment 4	14,901,788	CTATCTGCCACAAATGAATAAGGGCCTGG
FREM1 EcoRI Fragment 5	14,912,492	AGGCATTGGCTATGCCTTTCAGAGAATGC
FREM1 EcoRI Fragment 6	14,915,962	GCCACTGGCCTTATGCTGTATTCATTTCC
FREM1 EcoRI Fragment 7	14,924,529	CCCTGAATACTGGGAAGAGTGCTGATACCACAG
CER1 EcoRI Fragment 1	14,699,514	ACATCGTGGGAAGCCTCAGCCTTAGG
CER1 EcoRI Fragment 2	14,701,925	TGAGTTTGGTAGGTCTTGCTACCTGAAGCAGC
CER1 EcoRI Fragment 3	14,708,516	CAGGAGGATCAAAAAGGGTTAGGAGAACAGC
CER1 EcoRI Fragment 4	14,712,481	ACAGAGAGACTGTTCCAATGGGTGAAGATGG
CER1 EcoRI Fragment 5	14,721,023	AGGCAGGCATCATTCCTGTACTAATCAGGG
CER1 EcoRI Fragment 6	14,727,139	GGTGGAGTCAGAGGAAGATCAACCTTTCG
CER1 EcoRI Fragment 7	14,728,260	TCTCATGACTCTGTAAGGCTGGGGAAACC
CER1 EcoRI Fragment 8	14,736,783	CGTAGGTGCTCCTACCACTTTAGTCTGAGTGAAGC
ZDHHC21 Fragment 1	14,676,585	CGGTTAAGAGAGTCTCTGAAATGGGGAAGAAGG
ZDHHC21 Fragment 2	14,688,063	GTACCTCGTAGGCAAAAGTGACACGGTCC
ZDHHC21 Fragment 3	14,690,797	AGCTGTGGGTTTACAGGTAGGGCAATGACC
ZDHHC21 Fragment 4	14,691,752	TCAGTTCTGTCATTGGATTGCCAAGTCG
ZDHHC21 Fragment 5	14,695,787	AGGCAATCTCCCTCCACTATATCCTCCTCC
ZDHHC21 Fragment 6	14,699,514	GTTCACATGGATGCAACAGCAAATAGAGATGC
ZDHHC21 Fragment 7	14,701,924	GTGTGAGTTTGGTAGGTCTTGCTACCTGAAGC
NF1B EcoRI Fragment 1	14,299,060	GCTCTTTGCACTCTTTGCAAACTTGGAGC
NF1B EcoRI Fragment 2	14,300,723	CAAGGTCTTTCTCCTCATAAGCTCTCACTCAGC
NF1B EcoRI Fragment 3	14,305,724	CGTTCCTTTGACTTTGCTGTCAATGCTGC
NF1B EcoRI Fragment 4	14,310,338	TGTTACTCAGCATTAGGAAACAGTCACACTTGGG
NF1B EcoRI Fragment 5	14,319,007	TCTGCTCTCTGATTCAACAGTGGTAAATCCAGG

NF1B EcoRI Fragment 6	14,327,455	GAAGGCCTTCATATCACGAATGCAGAGATCC
NF1B EcoRI Fragment 7	14,334,074	GTCATTTTGAAGACCTTTGGCATCCATCTGG
SNAPC3 EcoRI Fragment 1	15,409,202	GGGTAGATGCCTCATGAATGGGATTAGAGTCC
SNAPC3 EcoRI Fragment 2	15,411,986	CTTGCTTTTGATGTCCACGTGCATGG
SNAPC3 EcoRI Fragment 3	15,413,722	GTTACATGGAGGCTAAGACCCAAATGACTGC
SNAPC3 EcoRI Fragment 4	15,416,858	CAGTGGGTAAGGGCAGGATCAGAATGG
SNAPC3 EcoRI Fragment 5	15,424,662	GGAACAAAGAGGGAGTGACACTTTTGACTCTTAGG
SNAPC3 EcoRI Fragment 6	15,431,783	CTTCCACATCTTTGTCTGTCCCTCTTTGGG
SNAPC3 EcoRI Fragment 7	15,440,453	AGACTATGGGAACCCACCACTGTGTTCAGC

**Luciferase Cloning Primers**	**Sequence (5’ to 3’)**
TTC39B alternative prom FOR	CCATGTGGTTGGAGCACAGTATATGC
TTC39B alternative prom REV	CCACACATCTGTTTCCCAACTGTACC
TTC39B PRE WT FOR	GTCCCTTTTTAGATAGGACATGAATCCCTGG
TTC39B PRE WT REV	CAGCTTAAGCATTCTAGGGATCTGTACC
TTC39B rs72700653 FOR	GAATCTGTTCAATAACATGACTTTTATTC
TTC39B rs72700653 REV	GAATAAAAGTCATGTTATTGAACAGATTC
TTC39B rs7874043 FOR	GCTATTTCTGAGGACACGCCACTTTACTAATC
TTC39B rs7874043 REV	GATTAGTAAAGTGGCGTGTCCTCAGAAATAGC
PSIP1 prom FOR	TGTTTGAGAGCCTGGGTACCACC
PSIP1 prom REV	TTCCTAATTCATTTCCAGTTGAGTCCC
CCDC171 prom FOR	CCTTTATTTCCTCTGGAGCAATTGTCC
CCDC171 prom REV	CGAGAACGCTGGCTCGGATTGGAAGAGC

**RACE and qRT-PCR Primers**	**Sequence (5’ to 3’)**
RACE-1	ACGAATTCTCGAGCCATGGCTTTTTTTTTTTTTTTN
RACE-2	ACGAATTCTCGAGCCATGGC
5RTTCExon3REV	GCAGAAATGCCGTTCTGGATGTCC
5RTTCExon2REV	GGTTTCCAAGGCATCTTCGAAAACG
3RTTCExon1BFOR1	CAACCGTTGGCTACATTTGATGG
qRTPCRTTCExon1AFOR	GGGAAATCGAGTAGCTGCACTCAGG
qRTPCRTTCExon1BFOR	CAACCGTTGGCTACATTTGATGG
qRTPCRTTCExon2REV	GCAGAAATGCCGTTCTGGATGTCC

Taqman Primers	Sequence (5’ to 3’)
TTC39B-202BFOR	TTGCTTTATTTGAACACGGTTCC
TTC39B-202BREV	GTGAGAAGATATTGAGATGGTTTCCA
TTC39B-202BFAM	AACGTCCTACACTTGTGCCA

EMSA Primers	Sequence (5’ to 3’)
rs72700653comFOR	AATCTGTTCAATAACATGACTTTTATTCA
rs72700653comREV	TGAATAAAAGTCATGTTATTGAACAGATT
rs72700653minFOR	AATCTGTTCAATAATATGACTTTTATTCA
rs72700653minREV	TGAATAAAAGTCATATTATTGAACAGATT
rs7874043comFOR	TATTTCTGAGGACAAGCCACTTTACTAAT
rs7874043comREV	ATTAGTAAAGTGGCTTGTCCTCAGAAATA
rs7874043minFOR	TATTTCTGAGGACACGCCACTTTACTAAT
rs7874043minREV	ATTAGTAAAGTGGCGTGTCCTCAGAAATA
AP1consensusFOR	CGCTTGATGACTCAGCCGGAA
AP1consensusREV	TTCCGGCTGAGTCATCAAGCG
FOXA1consensusFOR	CTGGTCTTAAAGGTGTTTACCTTGTCTGAT
FOXA1consensusREV	ATCAGACAAGGTAAACACCTTTAAGACCAG
Sp1consensusFOR	ATTCGATCGGGGCGGGGCGAGC
Sp1consensusREV	GCTCGCCCCGCCCCGATCGAAT
EMSAControlFOR	AGAGATTGCCTGACGTCAGAGAGCTAG
EMSAControlREV	CTAGCTCTCTGACGTCAGGCAATCTCT

ChIP-qPCR Primers	Sequence (5’ to 3’)
ChIPTTCPREFOR	ACGCAGACCCTGGAGTCTAA
ChIPTTCPREREV	GGGGGTTATGGTGAGAATCA
ChIPNegControlFOR	CAACCTGCACCTACCCAGTT
ChIPNegControlREV	GAACTCGAGACCAGCCTGAC

SUPPLEMENTAL METHODS

Patient Cohorts

Australian Ovarian Cancer Study (AOCS) ovarian cancer patients. The AOCS is a population-based, patient-control study that recruited 1859 women with ovarian, primary peritoneal or ovarian cancer from all Australian states between 2002 and 2006. Clinical data were collected from medical records at pre-specified time points: post-primary surgery/diagnosis, post-primary chemotherapy, at 12 months post diagnosis then at six monthly intervals to five years and annually thereafter. Information included age at diagnosis, primary site of tumor, histological subtype, FIGO stage and grade, residual disease following debulking laparotomy, type and dose of chemotherapy, time to first progression and vital status together with time to event variables. We selected 188 AOCS patients with extreme phenotypes for Phase 1: 94 patients with short PFS (median 10.6 months, 95% CI=[10.1,11.0]), despite having minimal residual disease (residual disease ≤1 cm), and 94 with relatively long PFS despite suboptimal debulking (residual disease >1 cm) (median 28.8 months, 95% CI=[24.5, 37.9]).

Mayo Clinic and The Cancer Genome Atlas ovarian cancer patients. To improve statistical power to detect novel loci associated with PFS, we performed a meta-analysis of GWAS results from AOCS and results from two further cohorts of patients with serous invasive ovarian cancer, from the Mayo Clinic (MAYO) and from The Cancer Genome Atlas (TCGA). The patients from MAYO were from a hospital-based series recruited between 2000-2008. The TCGA patients came from the pilot project, which was initiated in 2006 and for which data are publicly available (http://cancergenome.nih.gov/). The data from these two studies were combined for analysis (and are henceforth referred to as the 'MAYO+TCGA set'). Some patients were included in both studies, but these overlaps were detected so that each patient was included in the analysis only once. Apart from the selection on the basis of PFS and residual disease, the patients included from the MAYO and TCGA sets were subject to the same selection criteria as used for selecting AOCS samples.

Western analysis

Cells growing in 10 cm^2 tissue culture dishes were harvested with 0.25% trypsin and washed in phosphate buffered saline (PBS). Cell pellets were lysed in RIPA buffer [50 mM Tris-HCl, pH 8.0; 150 mM NaCl; 1% IGEPAL CA-630; 0.5% deoxycholate; 0.1% SDS; 1 mM DTT; protease inhibitor cocktail (Roche)] and clarified by centrifugation to remove cell debris. 20-40 micrograms of lysate supernatants were separated by SDS-polyacrylamide gel electrophoresis, electroblotted onto PVDF membranes by semi-dry transfer (Bio-Rad) and blocked in BLOTTO [5% dry milk powder; 0.1% Tween 20; PBS]. Sp1 was detected with144ng/ml rabbit anti-Sp1 monoclonal antibody (Cell Signaling), PSIP1 was detected with 100 ng/ml of rabbit anti-LEDGF/p75 polyclonal antibody (Bethyl Laboratories) and B-actin was detected with 400 ng/ml of rabbit anti-actin polyclonal antibody (Sigma-Aldrich). Primary antibodies were detected with horseradish peroxidase-conjugated goat anti-rabbit antibody (Cell Signaling). Detected proteins were visualized with enhanced chemiluminescence substrate (Bio-Rad) and the C-DiGit gel documentation system (LI-COR). Primary antibodies were detected with horseradish peroxidase-conjugated goat anti-rabbit antibody (Cell Signaling). Detected proteins were visualized with enhanced chemiluminescence substrate (Bio-Rad) and the LAS-500 gel documentation system (GE Healthcare).

5’ and 3’ Rapid Amplification of cDNA ends (RACE)
Five micrograms of total RNA was extracted from A2780 ovarian cancer cells using TRIzol and DNaseI (Life Technologies). For 5’RACE, cDNA was synthesized using 2pmol 5RTTCExon3REV and Superscript III (Life Technologies). cDNA was then column purified (Qiagen) and incubated with terminal transferase (New England Biolabs). PCR was performed using RACE-1 primer and 5RTTCExon2REV. For 3’RACE, cDNA was synthesized using 500ng RACE-1 primer and Superscript III. PCR was performed using RACE-2 primer and 3RTTCExon1BFOR1. Primers are listed in Supplementary Table 5 and all PCR products were sequenced verified by AGRF, Australia.

Gene expression analysis using quantitative real-time PCR (qRTPCR)
TTC39B-202 and *TTC39B-202B* mRNA levels were measured in A2780 and JAM ovarian cancer cell lines using qRTPCR and normalized against the housekeeping gene *TATA-binding protein* (TBP). All qRTPCRs were performed on a RotorGene 6000 using MyTaq HS DNA polymerase with the addition of 5 mM of Syto9, annealing temperature of 62°C and extension of 30sec. Analyses were performed in three independent experiments with each experiment quantified in duplicate. Primers are listed in Supplementary Table 5. TTC39B-202B mRNA levels in 18 cell lines and 149 AOCS tumors were measured using qRTPCR normalized against the housekeeping genes Beta Glucuronidase (GUSB) and Hypoxanthine Phosphoribosyltransferase 1 (HPRT1). Reactions were performed on an ABI7900HT Sequence Detection System (Applied Biosystems). Relative expression was calculated using the (delta-delta) Ct method. Primer and Probe sequences are listed in Supplementary Table 5. RNA was prepared from cell lines and tumor specimens using the RNeasy extraction kit (QIAGEN). RNA wasreverse transcribed with MMLV reverse-transcriptase (Life Technologies).

Taqman expression assays
Total RNA was extracted using Trizol (Life Technologies). Residual DNA contaminants were removed by DNAse treatment (Ambion) and complementary DNA was synthesized using random primers as per manufacturers’ instructions (Life Technologies). All qPCRs were performed on a RotorGene 6000 (Corbett Research) with TaqMan Gene Expression assays (Hs01045711_g1 for Sp1, Hs00916521_m1 for *PSIP1*) and TaqMan Universal PCR master mix. All reactions were normalized against *B-glucuronidase* (Catalogue No. 4326320E).

Kaplan-Meier (KM) plots
TTC39B expression data for 142 serous EOC tumors obtained through AOCS was derived by qRTPCR. Patients were grouped according to high or low expression as defined by dichotomisation around the upper decile of expression. Outcome and CCDC171 (C9orf93) expression data, generated by the Agilent microarray platform (probes A_23_P350658, A_24_P195081, A_32_P166478, A_32_P166480, A_32_P395879, A_32_P450587), for 374 patients, for whom information on treatment regimen was available, were extracted from The Cancer Genome Atlas (TCGA) interface (http://tcga-data.nci.nih.gov/) Nature 2011; 474:609-15. For either dataset, clinical outcome between groups was compared using Kaplan-Meier analysis followed by two-sided log-rank tests (SPSS v.22, IBM). Cox Proportional Hazards modelling (SPSS) was used to generate hazards ratios (HR) and 95% confidence intervals (95% CI)

SUPPLEMENTAL REFERENCES

1. Johnatty SE, Beesley J, Paul J, Fereday S, Spurdle AB, Webb PM, et al. ABCB1 (MDR 1) polymorphisms and progression-free survival among women with ovarian cancer following paclitaxel/carboplatin chemotherapy. Clinical cancer research : an official journal of the American Association for Cancer Research 2008;14:5594-601.
2. Rustin GJ, Vergote I, Eisenhauer E, Pujade-Lauraine E, Quinn M, Thigpen T, et al. Definitions for response and progression in ovarian cancer clinical trials incorporating RECIST 1.1 and CA 125 agreed by the Gynecological Cancer Intergroup (GCIG). International journal of gynecological cancer : official journal of the International Gynecological Cancer Society 2011;21:419-23.
3. Perren TJ, Swart AM, Pfisterer J, Ledermann JA, Pujade-Lauraine E, Kristensen G, et al. A phase 3 trial of bevacizumab in ovarian cancer. The New England journal of medicine 2011;365:2484-96.
